# 1,4-D-Glucan block copolymers: synthesis and comprehensive structural characterization

**DOI:** 10.1007/s00216-020-02388-z

**Published:** 2020-01-20

**Authors:** Payam Hashemi, Petra Mischnick

**Affiliations:** Institute of Food Chemistry, Technische Universität Braunschweig, Schleinitzstr. 20, 38106 Braunschweig, Germany

**Keywords:** Glucan, Cellulose ether, Transglycosylation, Block copolymer, Mass spectrometry

## Abstract

Multi-block glucans comprising permethylated and partially methylated blocks are compounds of interest. In order to monitor their formation by transglycosylation of corresponding starting glucans, a method has been developed and applied to model compounds. This method allows determining the average length of the blocks and the progress of incorporation of methyl blocks in partially methylated sequences with a random distribution. The method, comprising liquid chromatography mass spectrometry (LC-MS) and electrospray ionization collision-induced dissociation tandem mass spectrometry (ESI-CID-MS^*n*^) measurements of two types of peralkylated glucans representing derivatives of the target compounds, is comprehensively described and discussed. ESI-MS^*n*^ allows looking into the sequences of oligomeric domains. In addition, transglycosylation is followed by attenuated total reflection FTIR and NMR spectroscopy.

**Electronic supplementary material:**

The online version of this article (10.1007/s00216-020-02388-z) contains supplementary material, which is available to authorized users.

**Figure:**
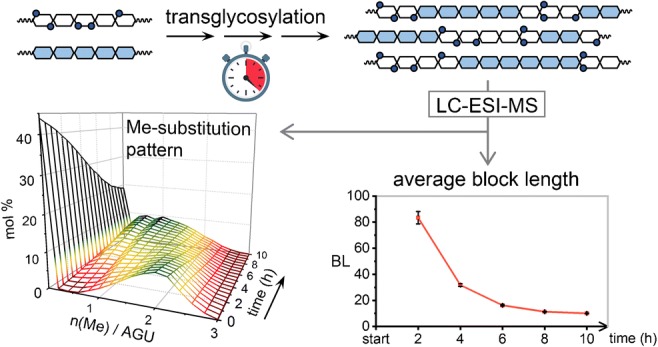
Graphical abstract

## Introduction

Originating from renewable resources, cellulose ethers are produced on a large scale by polymer analogous reactions, and they are one of the most important classes of cellulose derivatives with a wide range of applications in food, pharmaceuticals, and construction materials.

In addition to the type of the substituents, physicochemical properties of cellulose ethers strongly depend on the degree of polymerization (DP), degree of substitution (DS), and distribution of substituents along the polymer chains; this, per se, points out the importance of comprehensive analysis of substitution patterns for a better understanding of structure-property relationships [1] and improvement of modification processes.

Thermoreversible gelation of aqueous solutions of methylcellulose (MC) is strongly influenced by the methyl substituent pattern (methyl profile). For example, the gelation temperature of otherwise comparable methylcelluloses depends on the ratio of 2,3- to 2,6-di-*O*-Me glucosyl units [2]. Hydrophobic-hydrophobic interactions are held responsible for thermogelation; however, the true mechanism of gelation is not yet well understood. Kato et al. [3] considered tri-*O*-Me glucosyl units as cross-linking points for the gel network. Kobayashi et al. [4] suggested a two-step process, by which the hydrophobic association between highly substituted glucose units causes chain clustering, followed by phase separation–induced gelation. Studying the self-assembly of model diblock copolymers having unsubstituted glucose/cellobiose as hydrophilic heads and regioselectively methylated or permethylated glucose units as hydrophobic tails with a total DP of ≤ 28, Nakagawa et al. [5] showed the necessity of a sequence of at least 10 permethylated glucosyl units for gelation of methylcellulose to happen. Fibril formation prior to gelation was also observed and further studied using a systematic coarse-grained model [6–14]. Taking into account the role of highly methylated glucose units in thermogelation of methylcellulose, Adden et al. [15] suggested the cationic ring-opening polymerization of differently substituted cyclodextrins as a potential method for synthesis of glucan ether block copolymers having blocks of permethylated glucose units. Further mechanistic studies on model compounds, i.e., permethyl and perdeuteromethyl β-cyclodextrins, showed that the competing chain-transfer side reaction counteracted the cationic ring-opening polymerization from the early stages of the formation of block copolymers. As a result, upon consumption of cyclodextrins and elongation of copolymer chains, initially formed blocks were randomized rapidly and accompanied by a slight chain degradation [16]. Another hardship of this method was that copolymerization of permethyl cyclodextrin and perbenzyl cyclodextrin did not occur due to the different reactivities of the oxocarbenium ion intermediates. In a more recent study, Rother et al. [17] reported an unprecedented top-down approach for one-pot synthesis of glucan ether block copolymers by performing transglycosylation reaction (Trg) between cellulose derivatives, i.e., permethyl cellulose (per-MC) and perdeuteromethyl cellulose (per-Me-*d*_3_C) or perethyl cellulose. Upon Lewis acid–promoted cleavage of exoglycosidic oxygen, an active oxocarbenium ion is formed which undergoes an electrophilic attack at another glycosidic oxygen in an S_E_2-like manner to form a new glycosidic linkage while leaving another oxocarbenium ion behind to propagate the reaction. Additionally, re-combination and cross-combination of the ionic cleavage products has to be considered as well. The time-course study showed the randomization of blocks and the reduction of molecular weight (Mw) over the reaction time [17].

Rather than a glucan ether block copolymer with unsubstituted and permethylated blocks, our ongoing work aims at synthesis and characterization of glucan ether block copolymers comprising partially methylated and perdeuteromethylated blocks. Transglycosylation between benzyl-protected methylcellulose (BnMC) and permethyl cellulose, followed by the removal of protecting groups, produces the desired block copolymers.

Two main reasons for replacing unsubstituted blocks with partially methylated analogs were as follows: firstly, the water solubility of the partially methylated blocks (DS 1.89) in contrast to unsubstituted blocks, and the second reason was to level the difference in reactivity of starting materials in a transglycosylation reaction between perbenzyl cellulose and perdeuteromethyl cellulose. Previous work in our lab led us to the fact that the oxocarbenium ion of perbenzyl glucose has low reactivity in continuing the transglycosylation of linear glucans. In a mixed benzylated methylcellulose, four of the possible patterns have a methyl group at the position O-2, the most crucial position for the stability of the oxocarbenium ion. Therefore, it is expected that using benzylated methylcellulose allows the more reactive ones to drive the transglycosylation reaction forward.

On the other hand, Me blocks were replaced by their Me-*d*_3_ isotope in this study, only to facilitate the structural characterization of the blocky products. Of course, for further scale-up applications, the more economical methyl analog can be used in the same manner.

One of the challenges in this project was monitoring the reaction progress and quantitative characterization of such complex glucan ether block copolymer products comprising partially methylated and permethylated blocks. This paper focuses on this issue.

To obtain an ideal model for the characterization of these types of glucan block copolymers, model starting materials were synthesized and used in parallel transglycosylation reactions to develop an analytical method for monitoring the reaction progress and characterizing the products over the reaction time.

The structural characteristics of the products, i.e., DP, average block length, and distribution of blocks, are functions of time in transglycosylation reactions. Therefore, it is of high importance to carefully monitor the reaction progress and quench the reaction at the right time in order to obtain products with the desired composition, structure, and properties for targeted applications.

On account of the complexity of starting materials and products—both having mixed methyl substituents—chromatographic analyses hold no promise for reaction monitoring. Alternatively, reaction progress was monitored by comprehensive structural characterization of products over the reaction time by means of quantitative mass spectrometry which is a method of choice for analysis of these types of samples [1, 18].

By collision-induced dissociation tandem mass spectrometry (CID-MS^*n*^) of block copolymer products, the expected sequence of blocks in products could be proved.

## Materials and methods

### Materials and instrumentation

MC, TCI Chemicals, 7000–10,000 mPa·s 2% in water at 20 °C; cellulose acetate, Aldrich, 39.7 wt% acetyl, average *M*_n_ ~ 50,000 by GPC; methyl lithium (MeLi) solution, Aldrich, 1.6 M in diethyl ether; iodomethane (MeI) 99%, Sigma-Aldrich, stabilized with Cu; iodomethane-*d*_3_ (MeI-*d*_3_), Carl Roth, 99.5 atom% D, stabilized with Cu; dialysis membrane tubes, Carl Roth, MWCO 14,000; boron trifluoride diethyl etherate (BF_3_·Et_2_O), Aldrich, ≥ 46.5% BF_3_ basis. All reagents and solvents were used as received without further purification.

Attenuated total reflection Fourier transform infrared spectroscopy (ATR-FTIR) was carried out using a Bruker single-reflection Platinum ATR instrument. Spectra were processed by OPUS 7.0 software.

^1^H-NMR spectra were recorded at 20 °C on a Bruker Avance II 600 (600 MHz). Approximately 20 mg of the products was dissolved in 0.6 mL chloroform-*d* (99.8% D) containing 0.03% (v/v) SiMe_4_ (TMS) as the internal standard (Deutero GmbH, Kastellaun, Germany). Proton shifts (δ) are reported in parts per million (ppm) downfield from TMS.

### Synthesis of the starting materials

#### Per-MC and perdeuteromethyl methylcellulose

Per-MC (**1**) and perdeuteromethylated methylcellulose (Me-*d*_3_-MC, **3**) were synthesized by methylation and deuteromethylation of MC, respectively. Alkylation was performed using (Li-dimsyl) as the base, and MeI or MeI-*d*_3_ as the alkylating agents.

##### Li-dimsyl solution

To prepare 11 mL of 1.6 M Li-dimsyl, 11 mL of 1.6 M MeLi in Et_2_O was added to 11 mL anhydrous dimethyl sulfoxide (DMSO) at r.t. (22 °C) while stirring and purging nitrogen for 30 min to remove the Et_2_O residue.

##### Alkylation

One gram of methylcellulose (DS 1.89, 5.3 mmol, Mw ~ 189 g/mol per anhydroglucose unit (AGU)) was dissolved in anhydrous DMSO (30 mL). Freshly prepared Li-dimsyl solution (~ 11 mL, 3 eq./OH) was added dropwise to the solution of MC in DMSO. MeI or MeI-*d*_3_ (~ 1.1 mL, 17.2 mmol, 3 eq./OH) was added dropwise, and the reaction mixture was stirred for 24 h. Alkylation was repeated with half the amount of the reagents, and the solution was stirred for another 24 h. Thereafter, 4 mL MeOH was added and stirring continued for 20 min. The reaction mixture was transferred to a dialysis tube (MWCO 14,000) and dialyzed against water. After freeze-drying, 1.03 g (95%) per-MC and 1.05 g (93%) Me-*d*_3_-MC were collected. The disappearance of the OH vibration at around 3300–3500 cm^−1^ on the ATR-FTIR spectrum showed completeness of the reaction.

#### Per-Me-*d*_3_C

Per-Me-*d*_3_C (**2**) was synthesized by deuteromethylation of cellulose acetate (1 g, DS ~ 2.5, 3.74 mmol, Mw ~ 267 g/mol per AGU) in 30 mL anhydrous DMSO using freshly powdered NaOH (~ 2.2 g, 56.1 mmol, 5 eq. per OH/OAc) as base, and MeI-*d*_3_ (~ 3.5 mL, 56.1 mmol, 5 eq./OH) as the alkylating agent. Purification was performed as stated above. Complete alkylation was achieved by further deuteromethylation of the obtained product by Li-dimsyl and MeI-*d*_3_, in the same way as explained above to yield 0.75 g (94%) per-Me-*d*_3_C. For the IR, ^1^H-NMR, ESI-MS, LC-MS, and GC analysis of the starting materials, refer to the Electronic Supplementary Material (ESM) (Section 1).

### Transglycosylation reactions

#### Transglycosylation-a

One hundred two milligrams of **1** (0.50 mmol, Mw ~ 204 g/mol per AGU) and 107 mg of **2** (0.50 mmol, Mw ~ 213 g/mol per AGU) were added to a previously silylated 5-mL V-vial and dried over P_2_O_5_ under vacuum at 80 °C overnight. Five milliliters of anhydrous dichloromethane (DCM) was added to the mixture and let stir at 20 °C for 2 h. One milliliter of the reaction mixture was taken out, dried under the stream of nitrogen at 40 °C, and kept as a reference sample at the starting point of the reaction. Ten microliters of BF_3_·Et_2_O (0.08 mmol, 0.1 eq./AGU) was added to the rest of the reaction mixture. Nearly 1 mL of the reaction mixture was taken out every 2 h and quenched in 0.5 mL of 0.1 M NaHCO_3_. The organic phase was washed three times by deionized water and then dried under the stream of nitrogen at 40 °C. The collected amount of the samples at the start, 2 h, 4 h, 6 h, 8 h, and 10 h was 51.3 mg, 35.8 mg, 37.4 mg, 31.3 mg, 26.5 mg, and 17.6 mg, respectively (in total 199.9 mg, 95.7% of the added starting materials).

#### Transglycosylation-b

Transglycosylation-b was performed in parallel to transglycosylation-a (Trg-a). One hundred seven milligrams of **2** (0.50 mmol, Mw ~ 213 g/mol per AGU) and 104 mg of **3** (0.50 mmol, DS_Me_ ~ 1.89, Mw ~ 207 g/mol per AGU) were reacted in the same way as explained for transglycosylation-a. The collected amount of the samples at the start, 2 h, 4 h, 6 h, 8 h, and 10 h was 54.8 mg, 34.0 mg, 31.0 mg, 31.5 mg, 29.5 mg, and 24.8 mg, respectively (in total 205.5 mg, 97.7% of the added starting materials).

### Monomer analysis by gas-liquid chromatography

Monomer analysis of MC was performed as described by Voiges et al. [19]. Five independent samples (2 mg each) were taken. After total hydrolysis, alditol acetates were prepared by acetylation under alkaline conditions. Each sample was analyzed 3 times by gas-liquid chromatography flame ionization detector (GLC-FID) (total of 15 measurements). A final DS of 1.885 ± 0.002 was determined for the MC (ESM Fig. S7). Individual DS values for positions 2, 3, and 6 were DS(2) 0.78, DS(3) 0.42, and DS(6) 0.68, respectively.

#### GLC-FID analysis

Gas chromatography analysis was performed on a Shimadzu GC 2010, equipped with FID, Phenomenex Zebron ZB-5HT Inferno column (28.7 m), and a retention gap (methyl deactivated, 1.5 cm). Analysis conditions were as follows: H_2_ as carrier gas with a flow rate of 40 cm/s (linear velocity mode) and splitless injection at 250 °C. Temperature program started at 60 °C for 1 min, heating 20 °C/min up to 200 °C, 4 °C/min to 250 °C, and 20 °C/min to 310 °C and then remained constant for 10 min. Peak areas were corrected according to the molar effective carbon response (ECR) in FID [19]. Data evaluation was performed by GC solution software 2.41.00 (Shimadzu).

### Oligomer analysis by LC-MS

Nearly 3 mg of the starting materials or products was taken and partially hydrolyzed to short oligomers, followed by labeling with *m*-aminobenzoic acid (*m*-ABA). After dilution to 10^−4^ M and filtration through a syringe PTFE membrane filter (0.45 μm), the sample was analyzed 3 times by LC-MS [18, 20].

#### Partial hydrolysis

Each sample (~ 3 mg) was partially hydrolyzed by 1 M trifluoroacetic acid (TFA) in a 1-mL V-vial for 20 min at 120 °C. Repeated co-evaporation with toluene was performed at 22 °C under a stream of nitrogen to remove the TFA and to dry the sample.

#### Labeling with *m*-aminobenzoic acid

Partially hydrolyzed products were labeled with *m*-aminobenzoic acid by reductive amination in MeOH as described by Cuers et al. [20].

#### LC-MS analysis

For liquid chromatography mass spectrometry, the electrospray ionization ion trap mass spectrometer (ESI-IT-MS) (HCT Ultra ETDII; Bruker Daltonics, Bremen, Germany) was coupled to an Agilent LC system equipped with a binary pump (1100 Series) and a diode array detector (DAD) (G1315B). Mass spectra were evaluated using the data analysis 4.0 software (Bruker Daltonics, Germany).

Chromatography was performed using a reversed-phase RP-18 column (Phenomenex, Kinetex, 2.6 μm, 100 mm × 2.1 mm) with the mobile phases H_2_O/HOAc (99:1, v/v; A) and ACN/HOAc (99:1, v/v; B) in a linear gradient system (0 min (80 vol% A) and 35 min (10 vol% A)) and a flow rate of 0.2 mL min^−1^. Instrumental parameters were as follows: injection volume 10 μL, nitrogen as dry gas (10 L min^−1^, 365 °C) and as nebulizer gas (50 psi), capillary voltage − 4500 V, endplate offset voltage − 500 V, smart target 70,000, target mass 1000, compound stability 1000%, trap drive level 100%, scan range mode standard enhanced (8100 (*m*/*z*)/s), mass range 100–3000 *m*/*z*, and negative ion mode.

## Results and discussion

**Fig. 1:**
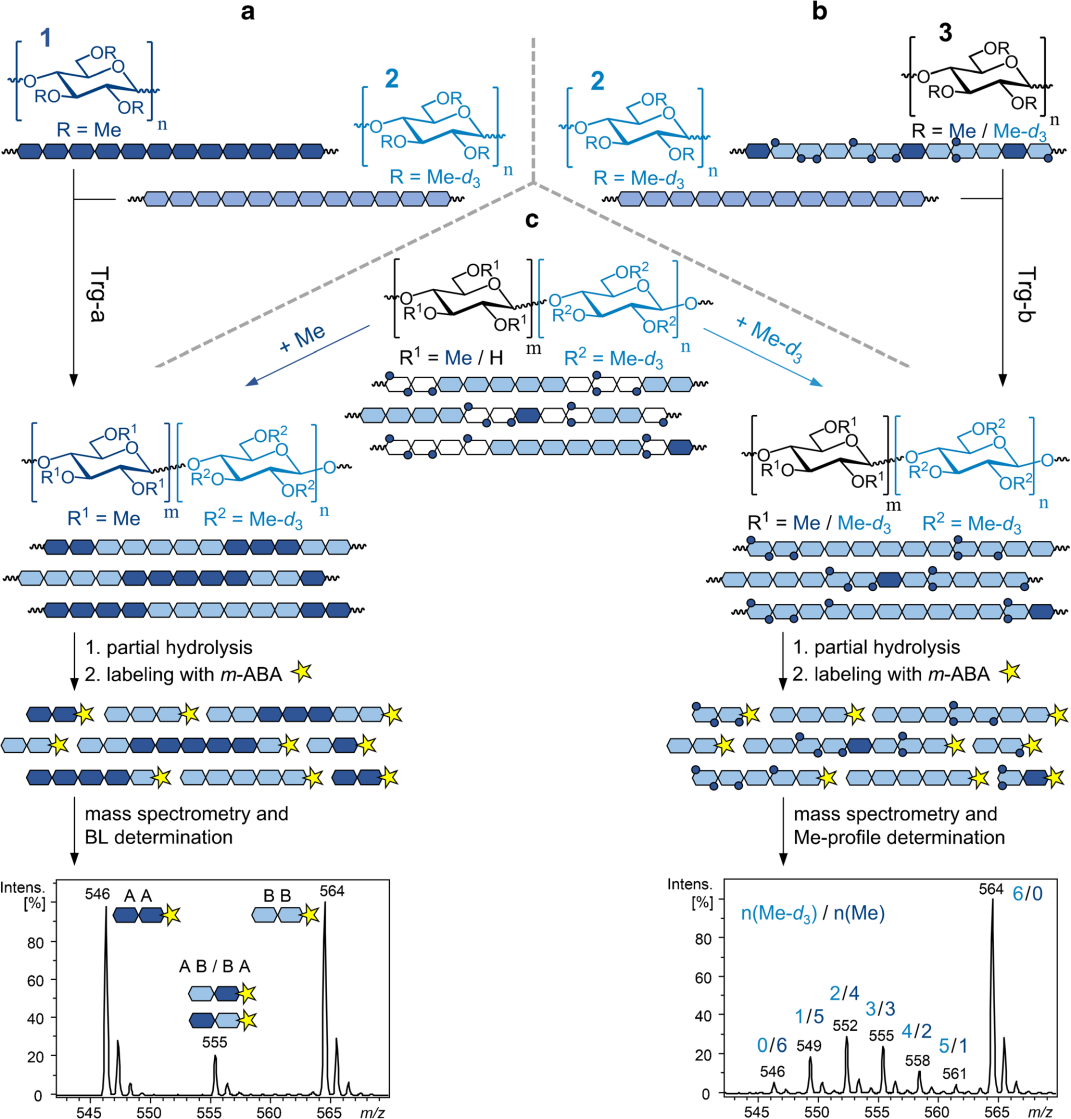
Schematic demonstration of (**a**) transglycosylation-a (Trg-a), (**b**) transglycosylation-b (Trg-b), and (**c**) the sample preparation and analysis method for structural characterization of glucan ether block copolymers by LC-MS

As mentioned in the “Introduction,” partially methylated blocks provide reactivity comparable with that of permethyl cellulose in transglycosylation reactions shown in Fig. 1. Methyl profile and average block length are two key factors in the structural characterization of such glucan ether block copolymers having both partially methylated and permethylated blocks. Determining the average block length and monitoring the changes of the methyl profile of such products by mass spectrometry is impaired by the coincidence of the *m*/*z* peak of the permethylated component in either block. Using deuteromethyl blocks instead of methyl blocks allows a much easier and more accurate determination of the average block length and methyl profile by quantitative mass spectrometry using the method presented in Fig. 1c.

By this method, each sample is divided into two halves. Deuteromethylation of one half produces a block copolymer product which is completely alkylated by isotopically distinct groups, i.e., Me and Me-*d*_3_. The similar chemistry of these isotope groups suppresses discriminations in electrophoretic mobility of the analytes in electrospray ionization, and thus, it is a prerequisite of quantitative mass spectrometry [1, 18]. The methyl substitution information in the partially methylated blocks is preserved and detectable by mass spectrometry in this portion (Fig. 1c, right).

Permethylation of the other half fills up the partially methylated blocks with methyl groups and thus produces block copolymers with two types of uniform blocks, i.e., per-Me and per-Me-*d*_3_ blocks. Each block and the block transition points produce one certain *m*/*z* peak on the mass spectrum, without any overlapping. Quantitative mass spectrometry of these products allows the determination of the average block length.

To prepare samples for mass spectrometry, products of parallel alkylation are partially hydrolyzed into short oligomers and then labeled by *m*-aminobenzoic acid.

Our ongoing research focuses on the synthesis of glucan ether block copolymers by performing a transglycosylation reaction between BnMC and per-Me-*d*_3_C, followed by debenzylation. The method mentioned above will be used for the structural characterization of the products. Obviously, for large-scale applications, the more economical permethyl analog (per-MC) can be used instead of per-Me-*d*_3_C.

In order to obtain a practical model for monitoring the transglycosylation reaction and characterizing the obtained glucan ether block copolymers, two parallel model transglycosylation reactions with model compounds were performed as shown in Fig. 1a and b. Transglycosylation between **1** and **2** produced block copolymers with per-Me and per-Me-*d*_3_ blocks. After partial hydrolysis and labeling with *m*-aminobenzoic acid, samples were analyzed by LC-MS. An exemplary MS spectrum of such products in the DP2 range is shown in Fig. 1a. Peaks with *m*/*z* 546 and 564 originate from the corresponding Me and Me-*d*_3_ blocks, whereas the one in the middle, *m*/*z* 555, indicates the alternating point of the blocks. For such block copolymers with isotope blocks, the average block length (BL) can be calculated by the following equation [15, 17, 18]:

$$ {\mathrm{BL}}_{\mathrm{DP}2}=\frac{\mathrm{Int}.\left(m/z546\right)+\mathrm{Int}.\left(m/z564\right)}{\mathrm{Int}.\left(m/z555\right)}+1 $$

In parallel, transglycosylation between **2** and **3** resulted in products with Me-*d*_3_- and mixed-substituted (Me/Me-*d*_3_) blocks. Samples were analyzed by LC-MS after sample preparation as mentioned above. An exemplary MS spectrum of such products in the DP2 range is shown in Fig. 1b. The number of Me and/or Me-*d*_3_ substituents of each peak is demonstrated above it.

**Fig. 2:**
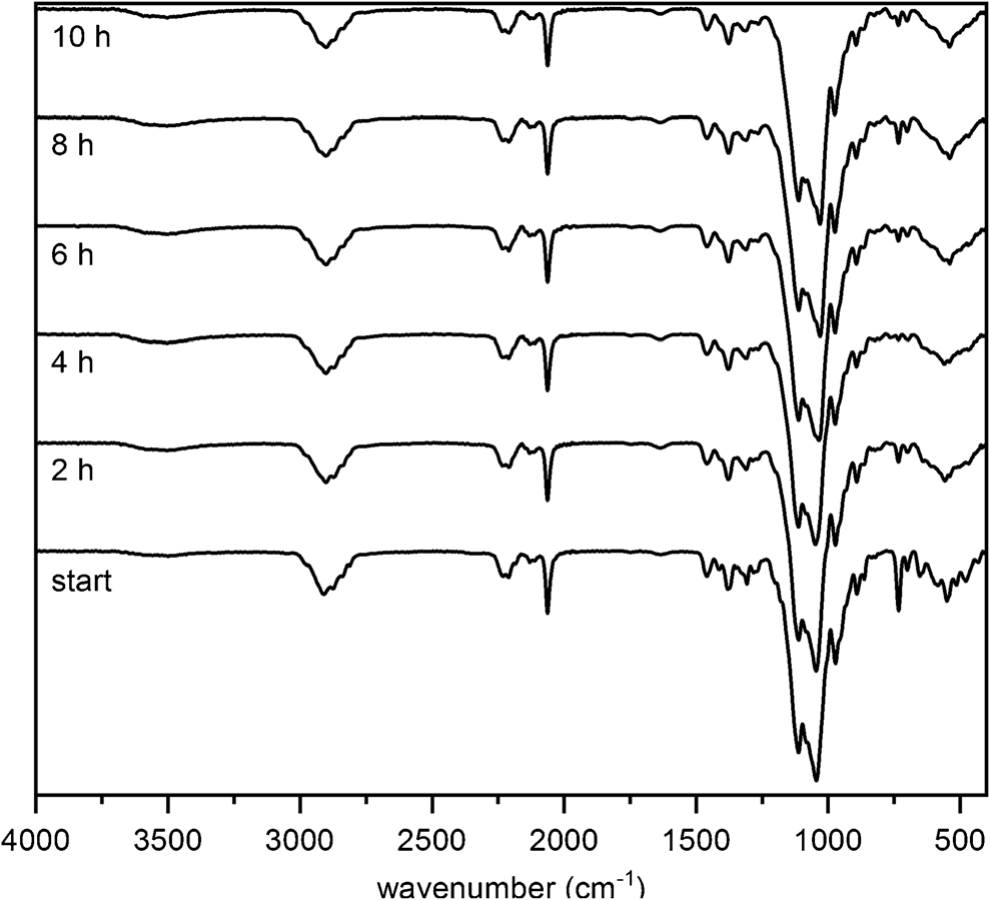
ATR-FTIR spectra of transglycosylation-b products

**Fig. 3:**
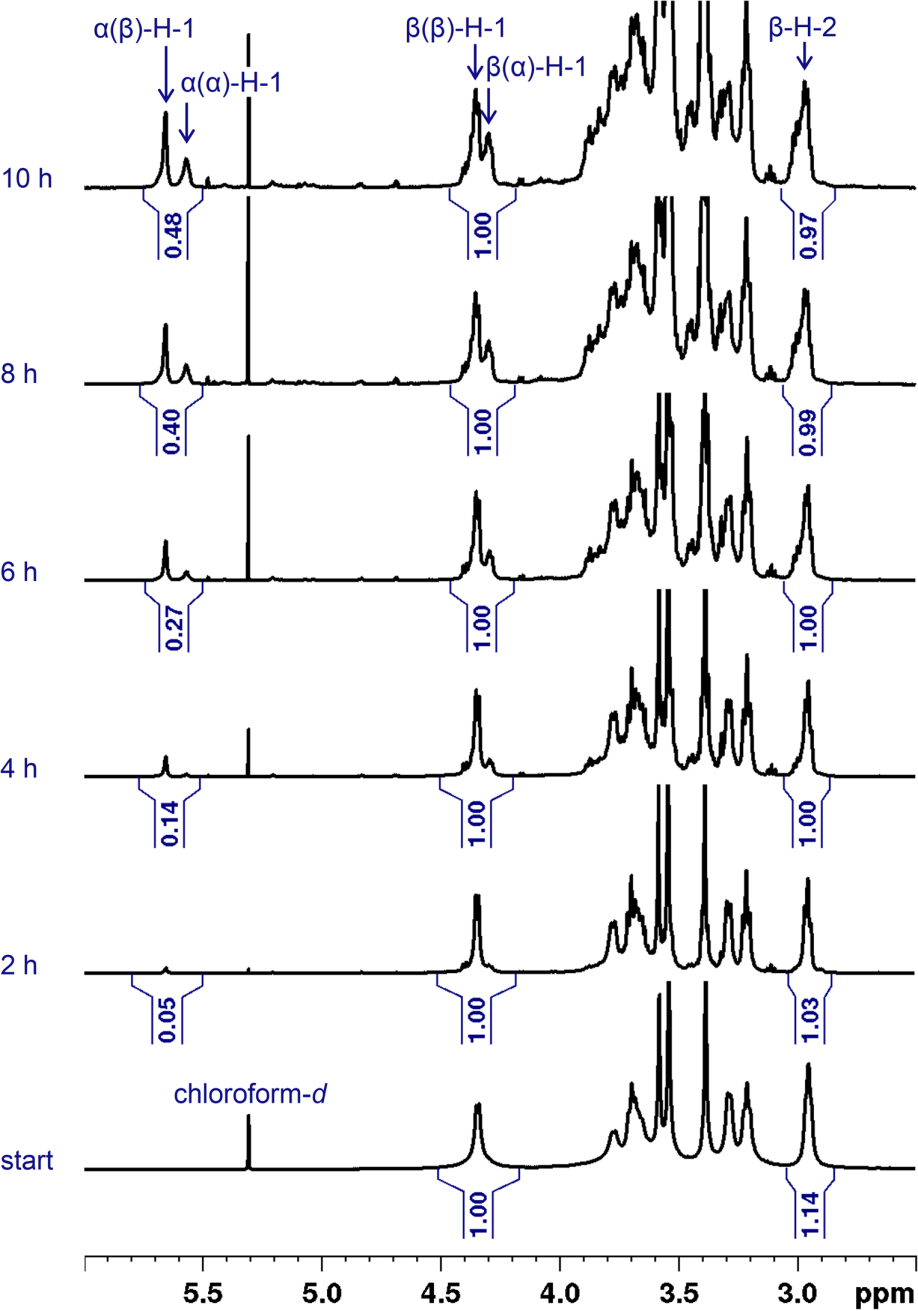
^1^H-NMR spectra of transglycosylation-b products (600 MHz, CDCl_3_)

For monitoring the transglycosylation reaction, a time-course study was performed by running the reaction for 10 h while taking samples every 2 h. After quenching and liquid-liquid extraction of the samples, IR and ^1^H-NMR spectra were recorded. Due to the similarity of the obtained spectra for these two parallel reactions, only the IR- and ^1^H-NMR spectra of the products of transglycosylation-b are shown in Figs. 2 and 3, respectively, whereas those of the transglycosylation-a are shown in ESM Figs. S8 and S9.

For both reactions, the IR spectra of the mixture of starting materials and the products are very similar. In case of a considerable chain degradation, which generates new terminal OH groups, a marked increase of OH vibration peak at around 3300–3500 cm^−1^ was to be expected from the early stages of the reaction. However, that was not the case in these two reactions (Fig. 2, ESM Fig. S8).

Starting materials of both reactions are cellulose ethers with β-1,4 glycosidic linkages. ^1^H-NMR spectra of the reaction products illustrate the gradual formation of α-1,4 glycosidic linkages over the reaction time. On the left shoulder of the H-1 signal of β-1,4–linked glucosyl units, a tiny doublet appears which might represent newly formed reducing end groups due to some chain degradation. Apart from that, there are no other vivid changes in the spectra (Fig. 3, ESM Fig. S9).

For structural characterization by mass spectrometry, i.e., determination of the average block length and methyl profile, reaction products were treated as shown in Fig. 1.

**Fig. 4:**
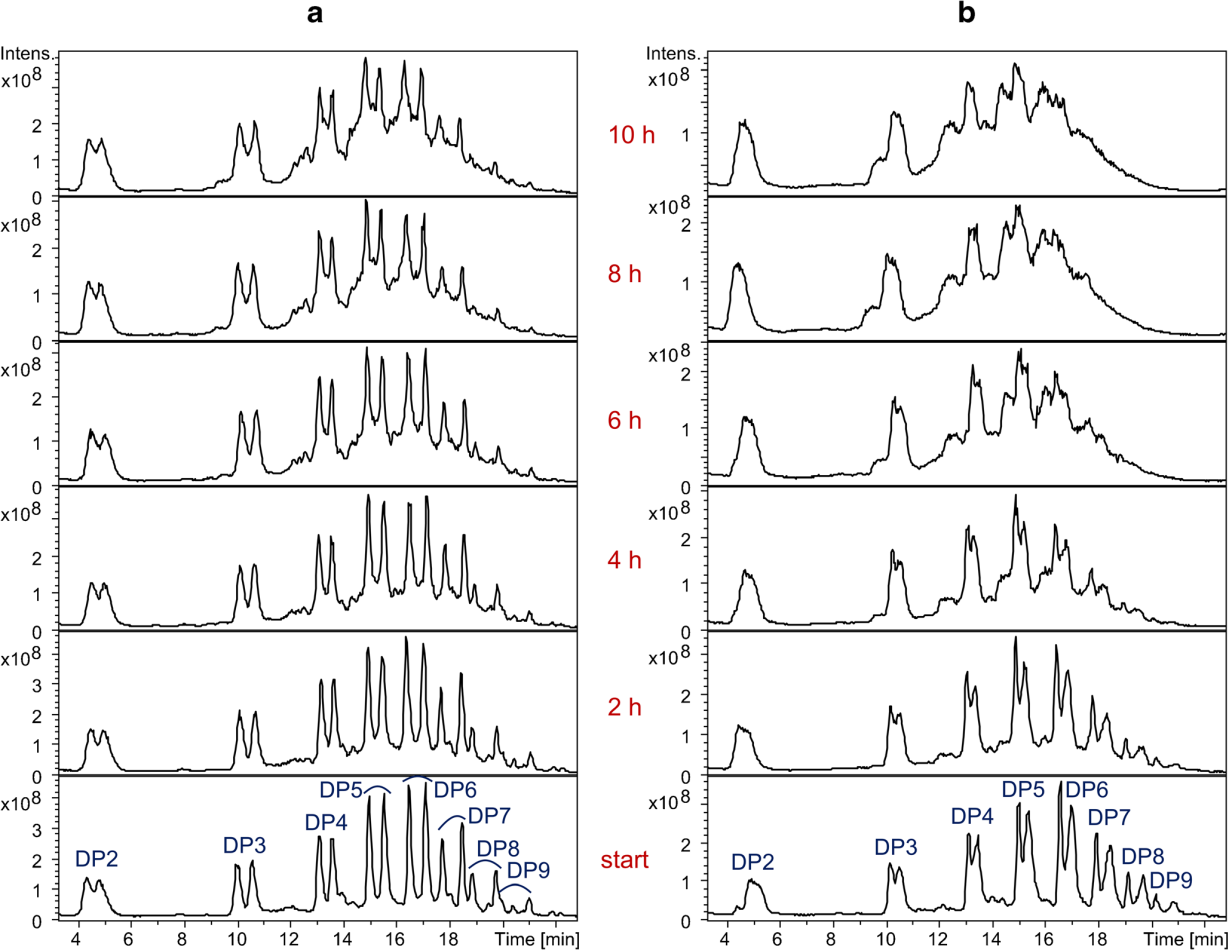
LC-MS total ion current chromatograms of the products of (**a**) transglycosylation-a and (**b**) transglycosylation-b prepared according to Fig. 1

LC-MS chromatograms of the obtained products from both transglycosylation reactions (partially hydrolyzed, and then labeled with *m*-amino benzoic acid) are demonstrated in Fig. 4. Even though the starting materials in both reactions are of similar nature, but with different isotopic substituents (i.e., Me, Me-*d*_3_), the LC-MS chromatograms are more complicated than the one presented by Cuers et al. [20] for a close to random Me-*d*_3_-MC derivative. These complications can be considered from various angles.

Looking at Fig. 4, the first point to consider is that under the conditions applied for LC-MS, not only the oligomers with various DP but also the isotopic components of each DP are separated. This explains the doublets for each DP on the total ion chromatogram (TIC). The order of elution of the isotopomer peaks is based on the fact that for a certain DP, components with more Me-*d*_3_ groups eluted faster than the Me analogs.

With increasing DP, the difference between the interactions of the isotopomers with the reversed phase becomes larger, and as a result, they are better resolved. This trend is obviously more easily observed for samples with the maximal difference between their blocks, i.e., Trg-a products. By more and more chemical mixing of the blocks as the reaction proceeds, the peaks are more poorly resolved. As shown in Fig. 4a, the separation of the isotopic doublet of DP9 of transglycosylation-a has grown so large that it has caused an overlap with the lagging isotopomer peak of DP8. Comparison of the LC-MS chromatograms in Fig. 4a and b clearly shows the influence of the sequence and configuration of the glucosidic linkages of the blocks, and the degree of chemical mixing of those blocks over the reaction time (including anomerization of the glycosidic linkages) on this phenomenon.

The second complication of the LC-MS chromatograms shown in Fig. 4 is the overlapping of peaks belonging to one DP as a consequence of these structural changes during the reaction progress. This can be obviously attributed to the formation of block copolymers from the starting materials. By gradual incorporation of the blocks into each other, the distinct chromatographic separation between them is lost. Furthermore, the small broad peaks which appear prior to the original peaks of each DP and gradually grow over time are in accordance with the formation of some α-1,4 linkages during the transglycosylation. An oligomer containing one α-linkage has no longer a linear chain conformation but a kink which is probably the reason for the less efficient interaction with the reversed phase.

The situation is different in Fig. 4b, which demonstrates the 1,4-glucooligosaccharides with per-Me-*d*_3_ blocks and mixed Me/Me-*d*_3_ sequences. Only the uniform per-Me-*d*_3_ shows a narrow peak at the beginning of the reaction which is gradually merged into the broad peak of the block with a random sequence. Here, also the peak shift to shorter retention times by formation of α-1,4-linkages can be recognized.

The third point to consider is that each of the starting materials has small amounts of under- and over-alkylated glucosyl units. Under-alkylation is a result of incomplete alkylation, whereas over-alkylation occurs due to alkaline degradation of glucan chains during the alkylation process, followed by alkylation of the newly generated terminal 4-OH groups. These under- and over-alkylated analytes have different polarities and are, thus, eluted at a different and wider time range. This adds to the complexity of the total ion current chromatogram even for the starting materials. With more and more chemical mixing of the materials, as the reaction proceeds, the chromatograms get more complicated.

**Fig. 5:**
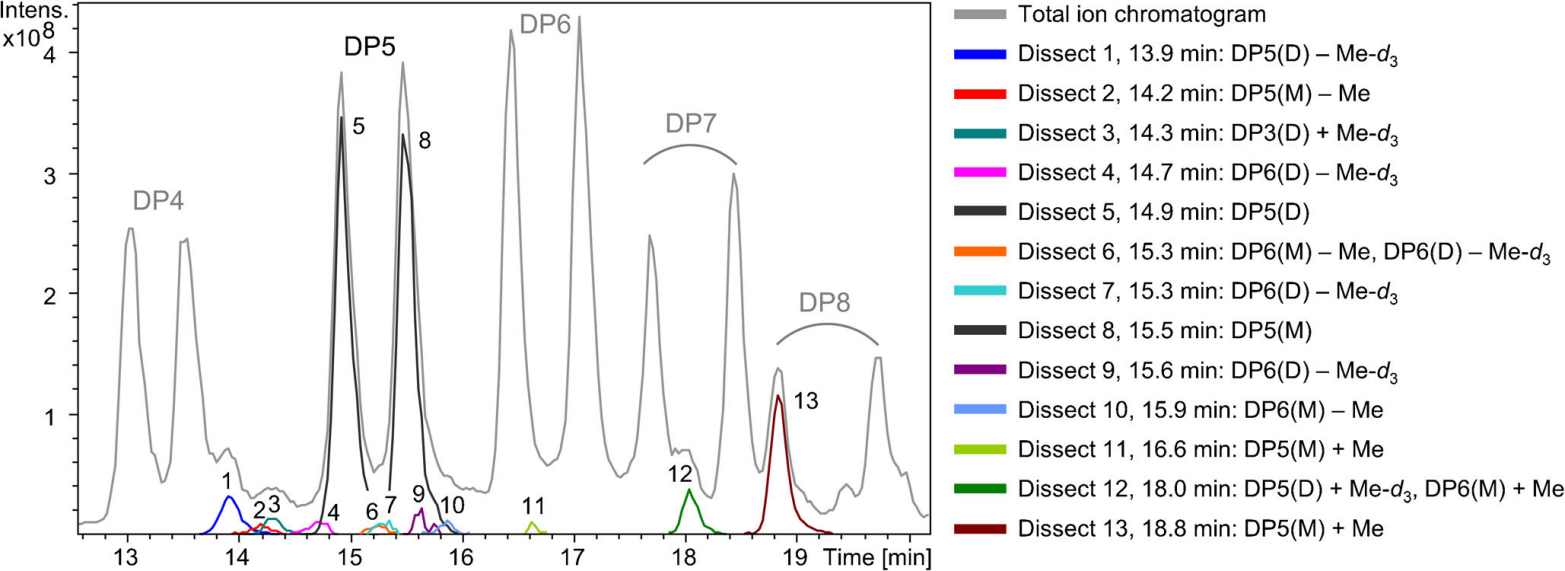
A cutout of total ion current chromatogram of transglycosylation-a at the start of the reaction, as well as the components of DP5 (main peaks and ± Me and Me-*d*_3_ peaks)

Figure 5 illustrates a cutout of the LC-MS chromatogram of transglycosylation-a at the starting point of the reaction, along with the dissected ion chromatogram components of DP5 and its under- and over-alkylated products, as well as under- and over-alkylated products of some other DPs which are overlapping with the main peaks of DP5. For the components of a certain DP, under-alkylated analytes elute earlier than main products on a reversed phase, and the over-alkylated analogs elute later.

**Fig. 6:**
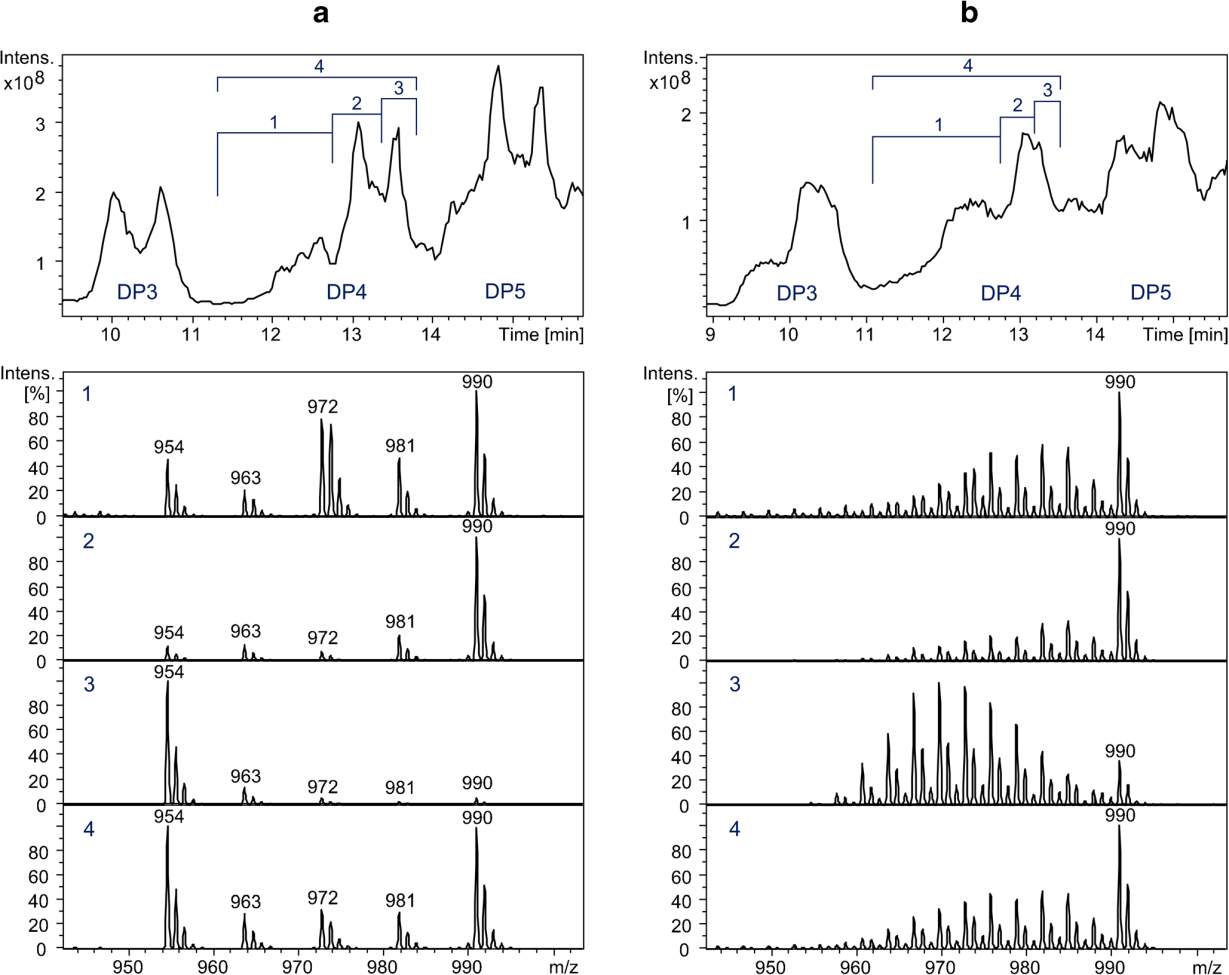
A cutout of total ion current chromatogram of the products of (**a**) transglycosylation-a and (**b**) transglycosylation-b after 10 h of the reaction, as well as the mass spectra produced by the integration of the demonstrated regions on the total ion current chromatogram of the LC-ESI-MS run

Figure 6 showing the cutouts of the LC-MS chromatograms of the products of transglycosylation-a and transglycosylation-b after 10 h (DP3–5), accompanied by the integrated mass spectra of the shown regions of DP4 on the total ion current chromatogram, proves the assignments discussed above.

According to the mechanism of transglycosylation reactions [17], it is statistically more probable to observe α-linkages at the change points of blocks than for a random glycosidic linkage within a block. The reason behind this statement lies in the fact that there has been, without a doubt, a cleavage and formation of a new glycosidic bond at each cross point, whereas not all glycosidic bonds within a block have experienced such cleavage and recombination. Since mixing of blocks and thus the occurrence of these change points proceeds with the progress of the reaction, the corresponding peaks on the LC-MS chromatogram of the products also increase (Fig. 3). The increase in the formation of the α-linkages is in agreement with ^1^H-NMR data (Fig. 3, ESM Fig. S9). Nonetheless, the ratio of α/β linkages does not affect the determination of the average block length and methyl profile by LC-MS, as long as a correct domain of the total ion current chromatogram is integrated for the production of the MS spectra and subsequent evaluations. This domain should comprise all the isotopic and conformational analogs of the analyte (spectrum 4 in Fig. 6).

Considering the points mentioned above, in order to obtain comparable LC-MS results for these types of samples which can be used for quantitative analysis, instrumental parameters of the mass spectrometer, such as the target mass, scan range, and scan speed, should not change during the analysis. Consequently, all the data presented in this manuscript are measured under constant LC-MS conditions as explained in the “Materials and methods” section.

**Fig. 7:**
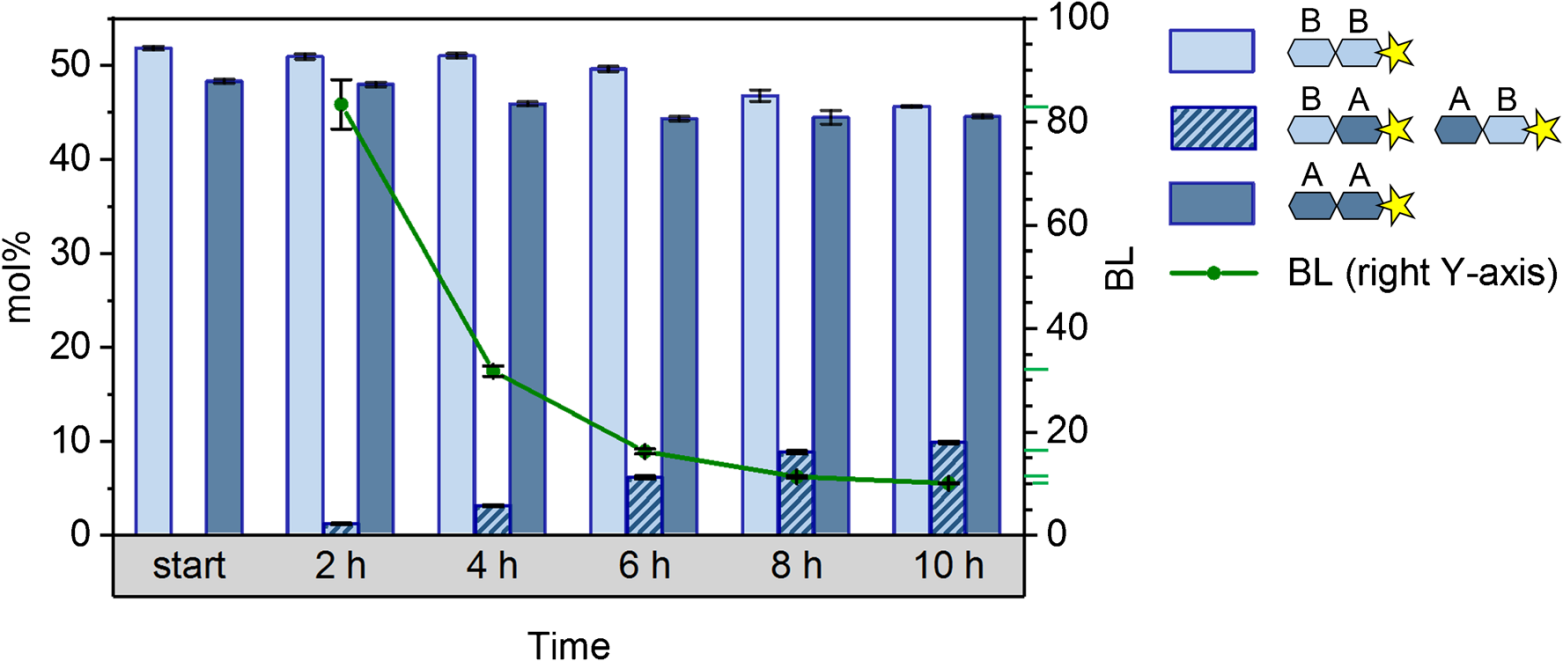
Evaluated average block length (BL) of transglycosylation-a products based on the LC-MS results at DP2 level: A: per-Me-AGU, B: per-Me-*d*_3_-AGU. Values are the averages of three times measurement of each sample

Figure 7 shows the evaluated LC-MS results of the products of transglycosylation-a at DP2 level. To simplify, glucose units of **1** and **2** are named A and B, respectively. At the starting point of the reaction, only the peaks of the starting homopolymer materials are visible (i.e., AA and BB). As the reaction proceeds and the blocks are chemically mixed, peaks of AA and BB decrease while the middle peak of AB/BA increases. The DP2 cutout of one of the LC-MS spectra of transglycosylation-a after 10 h which was used for the production of the corresponding data in Fig. 7 is depicted on the bottom left corner of Fig. 1. Using the equation mentioned earlier, BL of the products were evaluated and plotted on the right *y*-axis of the graph in Fig. 7.

During the optimization of the instrumental parameters for mass spectrometry analysis of transglycosylation-a and transglycosylation-b products, it came to our attention that for a given DP, the components with various Me/Me-*d*_3_ contents show slightly different responses to changes of the scan speed. This might be of importance in quantitative analysis of samples, such as transglycosylation-a products, which are, in fact, isotopomers. To investigate this observation, products of transglycosylation-a were analyzed by ESI-MS (syringe pump infusion) under identical conditions but different scan speeds. BL of the products were evaluated based on the acquired DP2 and DP3 results. Experimental details of this side study and the results are presented in ESM Section 3, Fig. S10. Further investigation of the influence of scan speed on quantitative ESI-MS and LC-MS analysis of glucan derivatives is in progress in our laboratory.

**Fig. 8:**
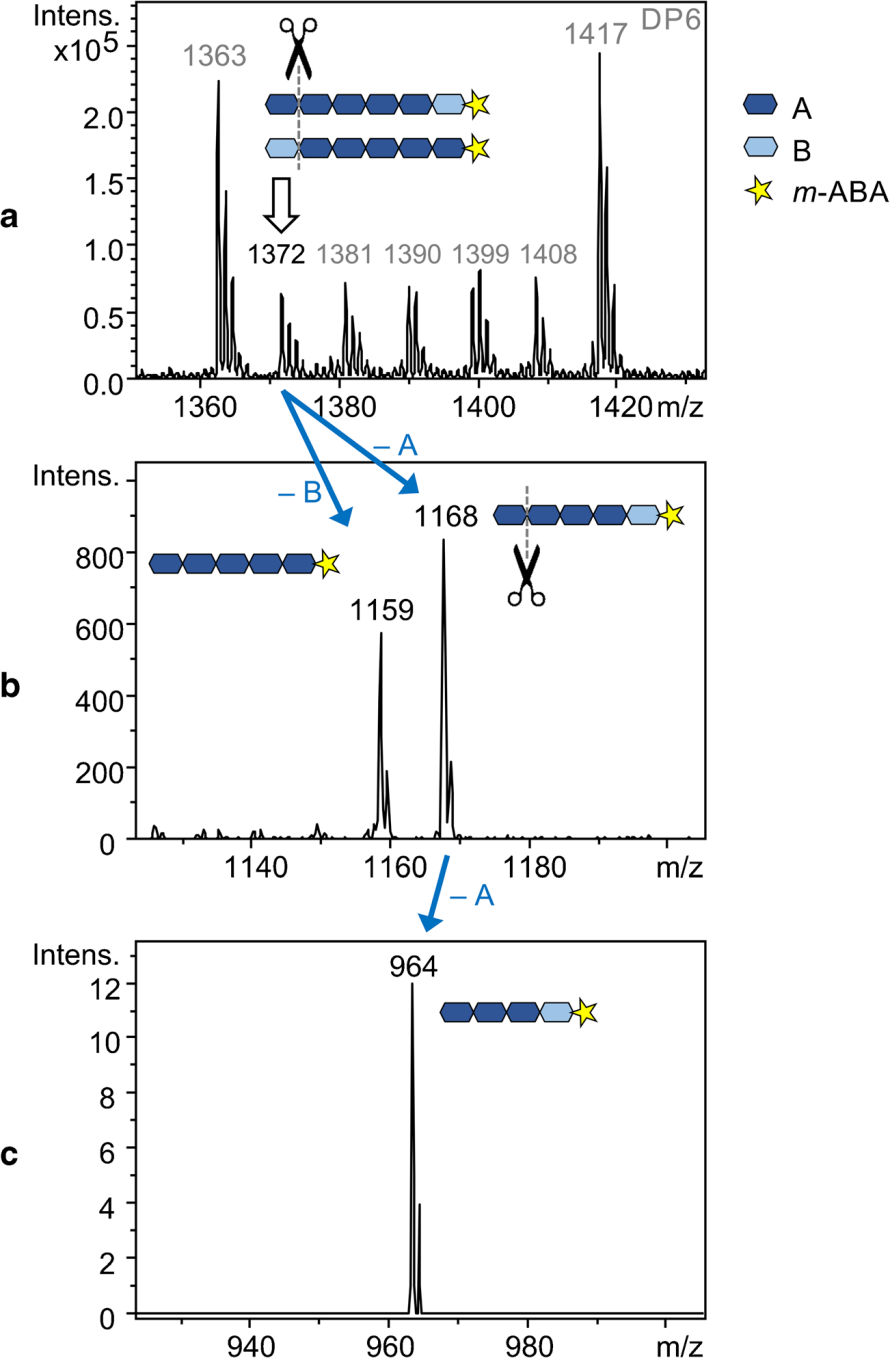
DP6 cutout of ESI-MS spectra of transglycosylation-a after 10 h (**a**), as well as the CID-MS^2^ (**b**), and CID-MS^3^ (**c**) of the indicated peaks

Figure 8 a illustrates the DP6 cutout of the ESI-MS spectrum of the products of transglycosylation-a at the reaction time of 10 h, as well as the schematic structure of the peak with *m*/*z* 1372, comprising five permethylated AGUs (A) and one perdeuteromethylated AGU (B). Fragmentation of this peak by CID-MS/MS produces two major peaks at DP5 level, i.e., *m*/*z* 1159 and 1168, which are the results of the elimination of one B or A from the non-reducing end of the oligomer, respectively (Fig. 8b). Further fragmentation of the peak with *m*/*z* 1168 (A_4_B) by CID-MS^3^, generated only one peak at DP4 level, i.e., *m*/*z* 964, which is the result of the elimination of AA from the non-reducing end of the oligomer with DP6 (Fig. 8c). This indicates that no single B unit is found in the sequence of DP6, but there is only one A–B transition. Since a single B should occur in positions 2, 3, 4, and 5 of DP6 with the same probability, these alternative sequences can also be excluded. A similar strategy was applied to the middle peak with *m*/*z* 1390 in Fig. 8a, showing that for the 20 possible combinations of A_3_B_3_, only the AAABBB and BBBAAA sequences exist at detectable amounts. The results are demonstrated in ESM Fig. S11. Three major conclusions can be drawn from the data illustrated in Fig. 8 (and ESM Fig. S11).

This sequence analysis presents a perspective for average block length analysis on a higher DP level than DP2 (as presented in Fig. 7). Not only it confirms the blockwise structure of transglycosylation products even after 10 h of reaction, but also it adds a higher degree of certainty to the evaluated average block lengths shown in Fig. 7. To elaborate, if the sample considered here (product of transglycosylation-a after 10 h) with an average block length of 10 already includes isolated unit A or B or short blocks (Fig. 7), more than one peak would be expected in the CID-MS^3^ spectrum in Fig. 8c. The more or less equal amounts of all transition products with DP6 (Fig. 8a) also indicate that these are diblock oligosaccharides occurring with the same probability after hydrolysis. Sequences of ≤ 5 units of A or B can be neglected at this average block length.

The above-stated fact shows that the performed transglycosylation reactions have followed a statistic-based mechanism. In case glycosylation was preferred at the chain ends (because of greater freedom of the polymer chains or better accessibility of the glycosidic bonds), more chemical mixing would occur near the chain ends. That would result in shorter block lengths at chain ends and longer block lengths at the inner parts of the chains; thus, a wide distribution of the average block length and inconsistent mixing of blocks would be expected.

The third point to consider is that the presence of both A_5_B-*m*-ABA and BA_5_-*m*-ABA shows that both of the starting materials have undergone the transglycosylation reaction to form block copolymer products. Alternatively put, the oxocarbenium ions formed by the cleavage of each of the starting materials were active enough to attack other glycosidic bonds of the other starting materials and form the block copolymers. This is, indeed, no surprise in such a transglycosylation reaction between materials with isotope Me and Me-*d*_3_ groups—which have more or less identical chemical properties. Materials with different substituents, however, demonstrate different reactivities in transglycosylation reactions. In that case, the abovementioned sequence analysis technique can provide a profound insight into the relative reactivity of the starting materials to react with each other in a transglycosylation reaction to form block copolymer products. An example of such a reaction—which is currently under investigation in our lab—is transglycosylation between BnMC and **2** which was briefly explained in the “Introduction.”

**Fig. 9:**
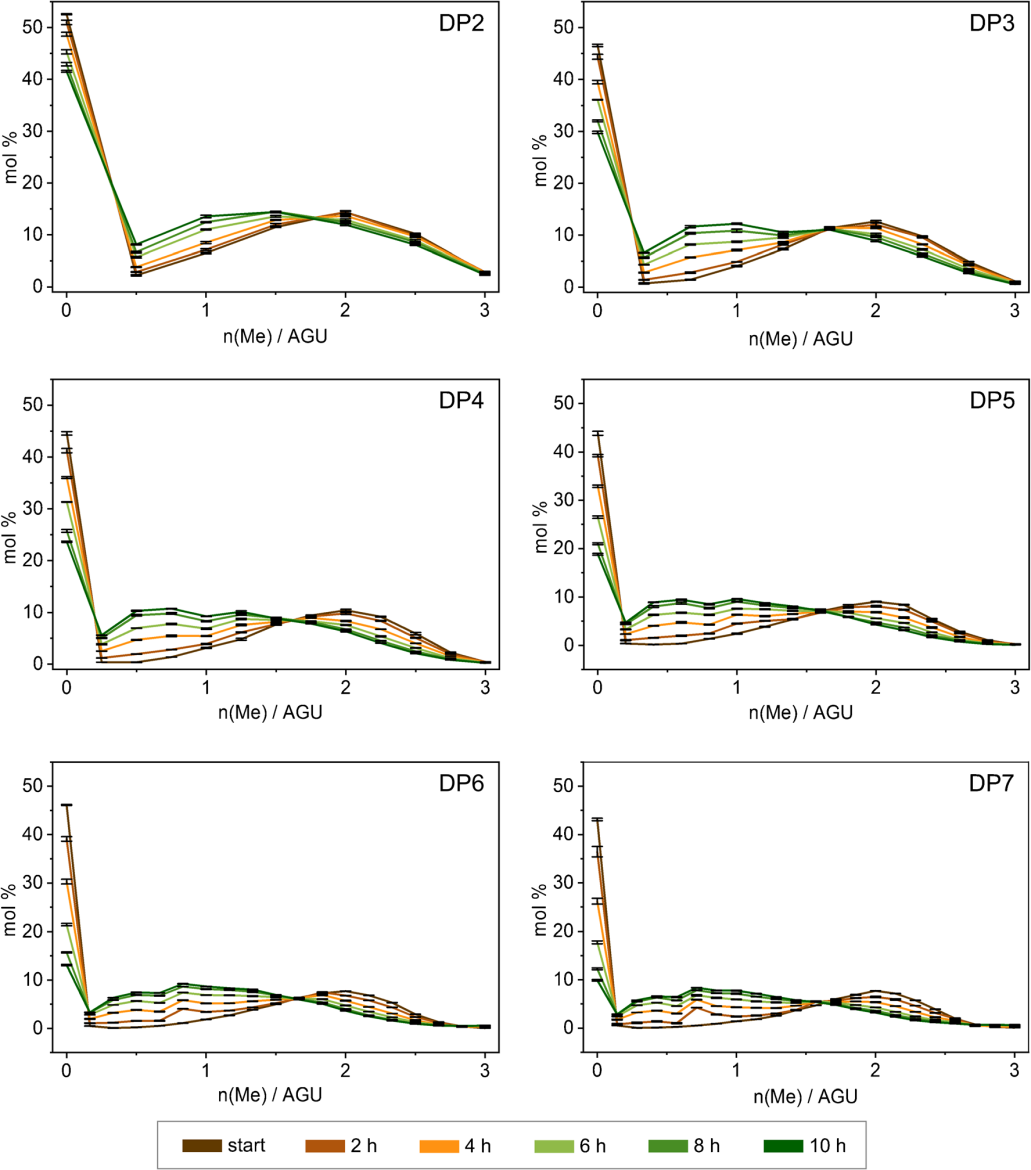
Evaluated methyl profiles of transglycosylation-b products based on the LC-MS data at DP2–7 levels. Values are the averages of four times measurement of each sample

Figure 9 presents the evaluated methyl profile of transglycosylation-b products at DP2–7 levels. The DP2 cutout of one of the LC-MS spectra of transglycosylation-b at the start of the reaction which was used for the production of the corresponding methyl profile in Fig. 9 is depicted on the bottom right corner of Fig. 1. Methyl profiles in Fig. 9 are mirrored depictions of their MS spectra because it is customary to plot the methyl profile versus the increasing number of Me groups per AGU on the *x*-axis. In case the results are representative of the applied material, the DS evaluated for each profile of each DP should be the average of the DS of the starting materials which were used in equimolar ratio, thus 0.95 for Me and 2.05 for Me-*d*_3_. This value was matched with a slight deviation for DP2–7 (average for all DPs and reaction times is 0.97 ± 5%).

Following the course of the reaction by looking at the DP2 graph in Fig. 9, it is evident that the highest peak which mainly originates from **2** decreases as it incorporates into the other starting material (i.e., **3**). On the other hand, the methyl profile of **3** gradually shifts toward lower methyl content values.

One reason for using **2** instead of its more economical analog (**1**) was for better distinguishing between blocks originating from MC and those from fully alkylated cellulose. A further advantage in the transglycosylation-b model experiment was that the peak of **2** was located far from the apex of the methyl profile curve of **3** on the methyl profile graph.

A more pronounced reduction of the peak 0 Me/AGU (i.e., 15 Me-*d*_3_) on the graphs shown in Fig. 9 with increasing DP is due to statistical reasons. Interesting is the development of a polymodal methyl profile for DPs higher than DP2 (for a better demonstration of this trend, refer to ESM Fig. S12). It looks as a secondary profile is pursuing the main profile at a fixed distance. This polymodality can be mainly attributed to statistically rooted reasons. In contrast to random methylation of a chain, here peralkylated sequences are incorporated into random sequences. Moreover, there are other probable reasons which might play minor parts. For instance, a probable reason for this polymodality can be the overlapping of the methyl profile of the main products with the methyl profile of the over/under-deuteromethylated products—which are the result of transglycosylation of the under- and over-deuteromethylated parts of the starting materials, the latter at terminal 4-OH.

To further investigate this theory, we tried to deliberately eliminate the contribution of over/under-deuteromethylated products from the graphs to see whether this would eliminate the secondary profile. Therefore, the LC-MS results had to be re-evaluated in a certain way. The experimental details and results of this side study are presented in Online Resource Section 5.2. In short, by this exercise, the polymodality of the methyl profiles was slightly faded but did not completely disappear. This confirms the observed polymodality which is expected as long as the incorporated blocks are not completely randomized.

This reaction was performed to provide a model for a better understanding of the transglycosylation reaction by monitoring the methyl profile of the products over the reaction time. Accordingly, the change of each *n*(Me)/AGU was plotted against time for DP5 (the procedure is, of course, applicable to other DPs as well). By fitting the plotted experimental data using the Boltzmann sigmoidal formula (ESM Section 5.3), a realistic curve for each *n*(Me)/AGU against time was obtained. By combining all these data, a model for evaluation of the methyl profile of transglycosylation products as a function of time was obtained. This model is based on the experimental data and is valid only within the applied time range of the reaction. In other words, extrapolation to longer reaction times is not possible by this model.

It is worth pointing out that from the practical point of view, the reaction time of the performed transglycosylation reactions was long enough to allow considerable mixing of blocks to occur, and also a proper number of samples were taken to monitor the reaction progress. The concept of developing such a model is of significant value for transglycosylation reactions between materials that require much longer reaction times, where it is not practically desirable to take samples every 2 h and analyze them all. In that case, it is enough to perform the reaction once and take a few samples to produce such a model for methyl profile of the products during that particular reaction. Thereafter, based on the obtained model, one can decide the right time to quench the reaction in order to get the desired product.

**Fig. 10:**
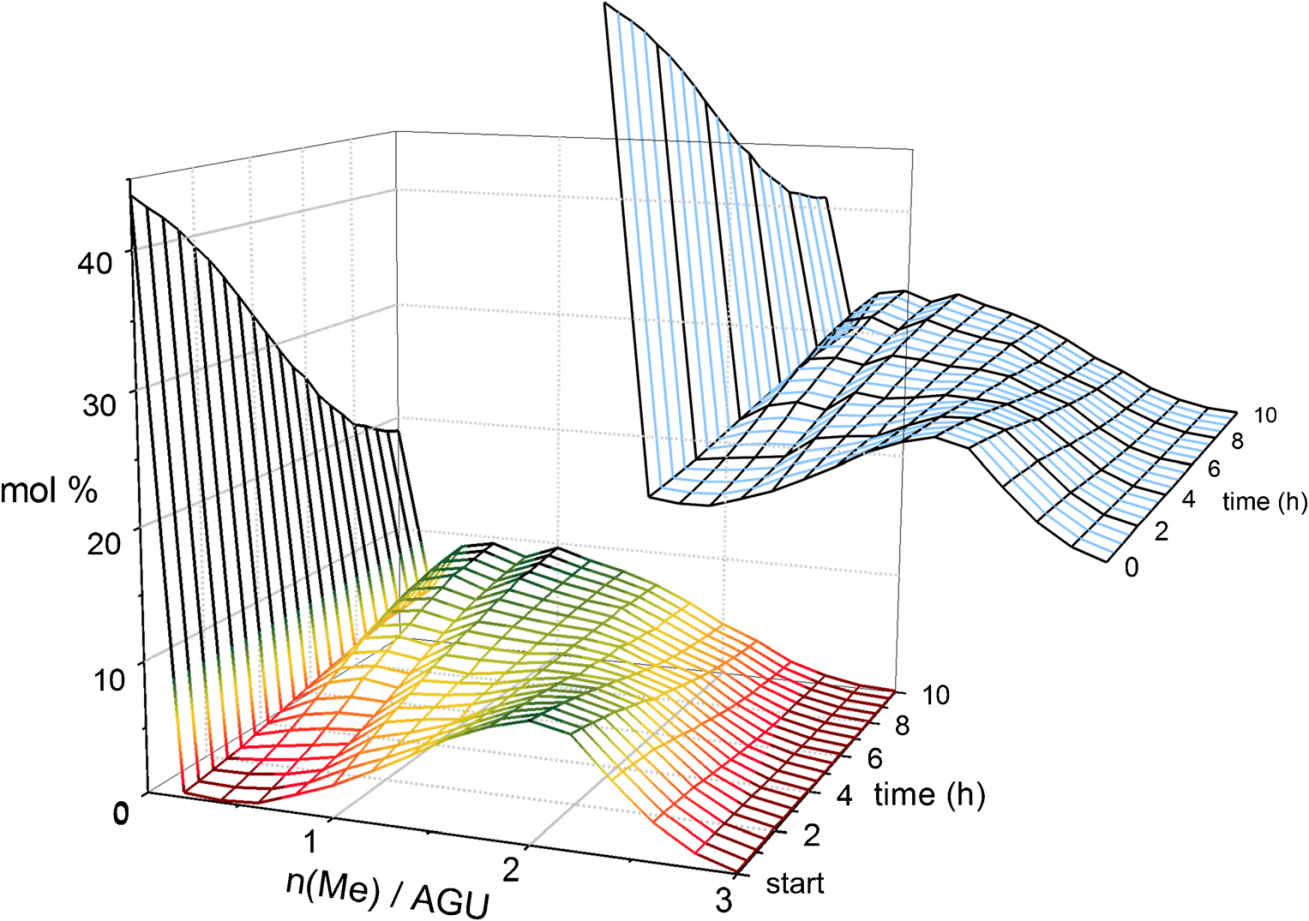
Simulated methyl profile of the products of transglycosylation-b over the reaction time. The black methyl profiles in the same upper graph show the reaction times at which experimental data are available, whereas the blue ones are simulated by the model

Figure 10 illustrates a simulated colored 3D construction of methyl profile of transglycosylation-b products over the reaction time at DP5 level. The same upper graph in two colors is depicted as a guide to show that there are experimental data for the methyl profiles in black at the beginning and every 2 h of the reaction, whereas the blue ones are simulated by the model obtained as explained above for every 30 min in between the experimental data. Production of the model for DP5 is explained in more detail in ESM Section 5.3.

## Conclusion

It has been shown that the formation of multi-block glucans by transglycosylation of protected methylcellulose and fully methylated cellulose could be monitored by using the isotopomeric per-Me-*d*_3_ cellulose and filling the OH of the partially methylated domains (after deprotection) with Me and in parallel with Me-*d*_3_ groups. By such sample preparation, on the one hand, glucans with uniform Me and Me-*d*_3_ blocks are formed from which the average block length can be quantified. On the other hand, the perdeuteromethylated products maintain the methyl pattern of the partially methylated domains and allow monitoring of the integration of per-Me-*d*_3_ sequences into these regions. The time course of this integration could be illustrated by fitting the plotted experimental data over (0–10 h) using the Boltzmann sigmoidal formula. ESI-CID-MS^3^ measurements of the isolated oligoglucans of DP6, obtained by partial hydrolysis of the products, proved that at an average BL of 10, no isolated or short domains of ≤ 4 glucosyl units can be detected. This is important for cooperative interaction of the target cooperative interaction of the target compounds which are presently prepared in our lab.

## Electronic supplementary material

ESM 1 (PDF 3.19 mb)
