# Supplementary material for: 1,4-D-Glucan block copolymers: synthesis and comprehensive structural characterization
Source: Anal Bioanal Chem. 2020 Jan 20;412(7):1597–610. doi: 10.1007/s00216-020-02388-z (PMC7026270; doi:10.1007/s00216-020-02388-z)
Supplement: Supplementary file 1 — (PDF 3.19 mb) [file 216_2020_2388_MOESM1_ESM.pdf]

Analytical and Bioanalytical Chemistry

Electronic Supplementary Material

1,4-D-Glucan block copolymers: synthesis and comprehensive structural characterization

Payam Hashemi, Petra Mischnick

1. Characterization of the starting materials

1.1. ATR-FTIR spectroscopy

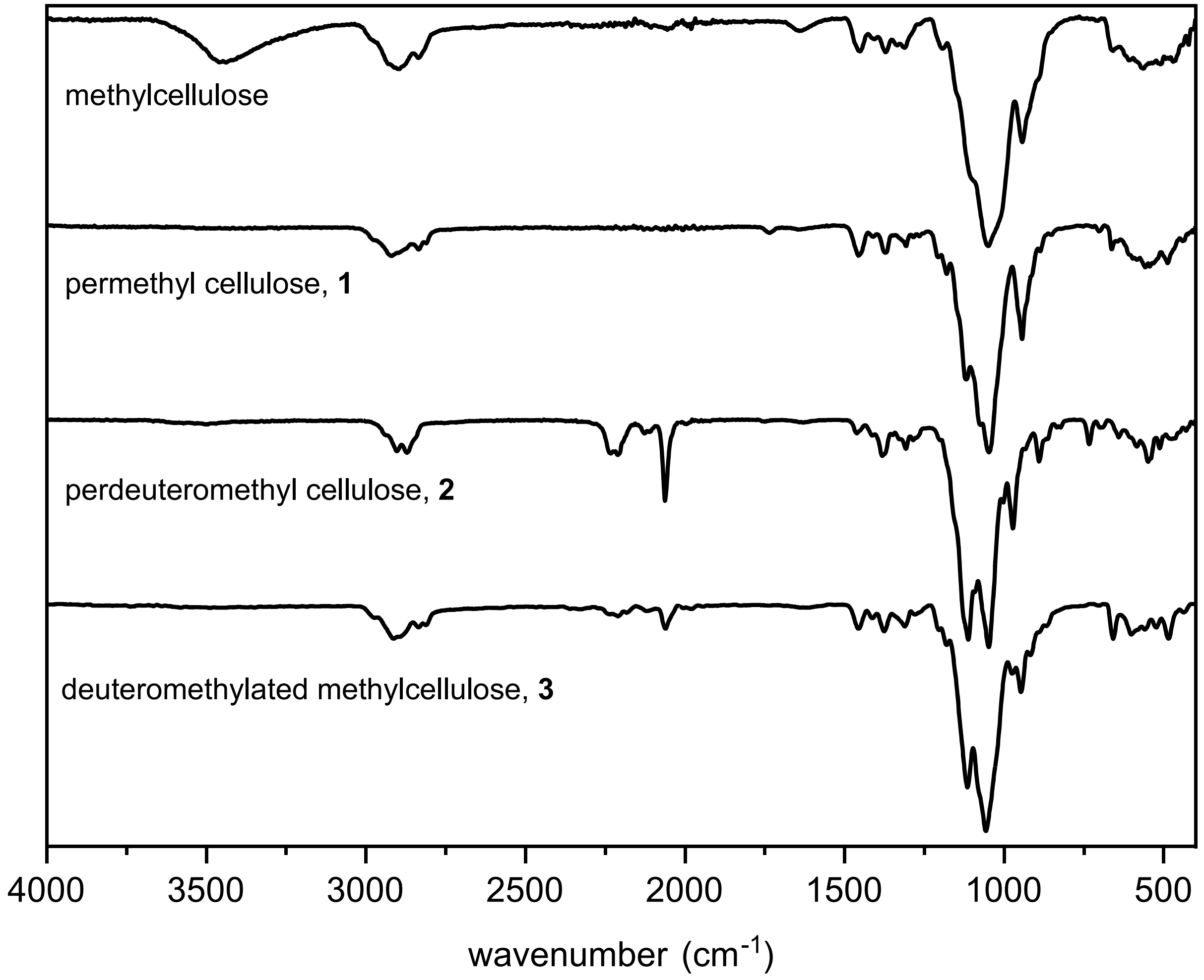

Fig. S1 ATR-FTIR spectra of **1**, **2**, **3**, and MC (for comparison)

1.2. ^{1}H-NMR spectroscopy

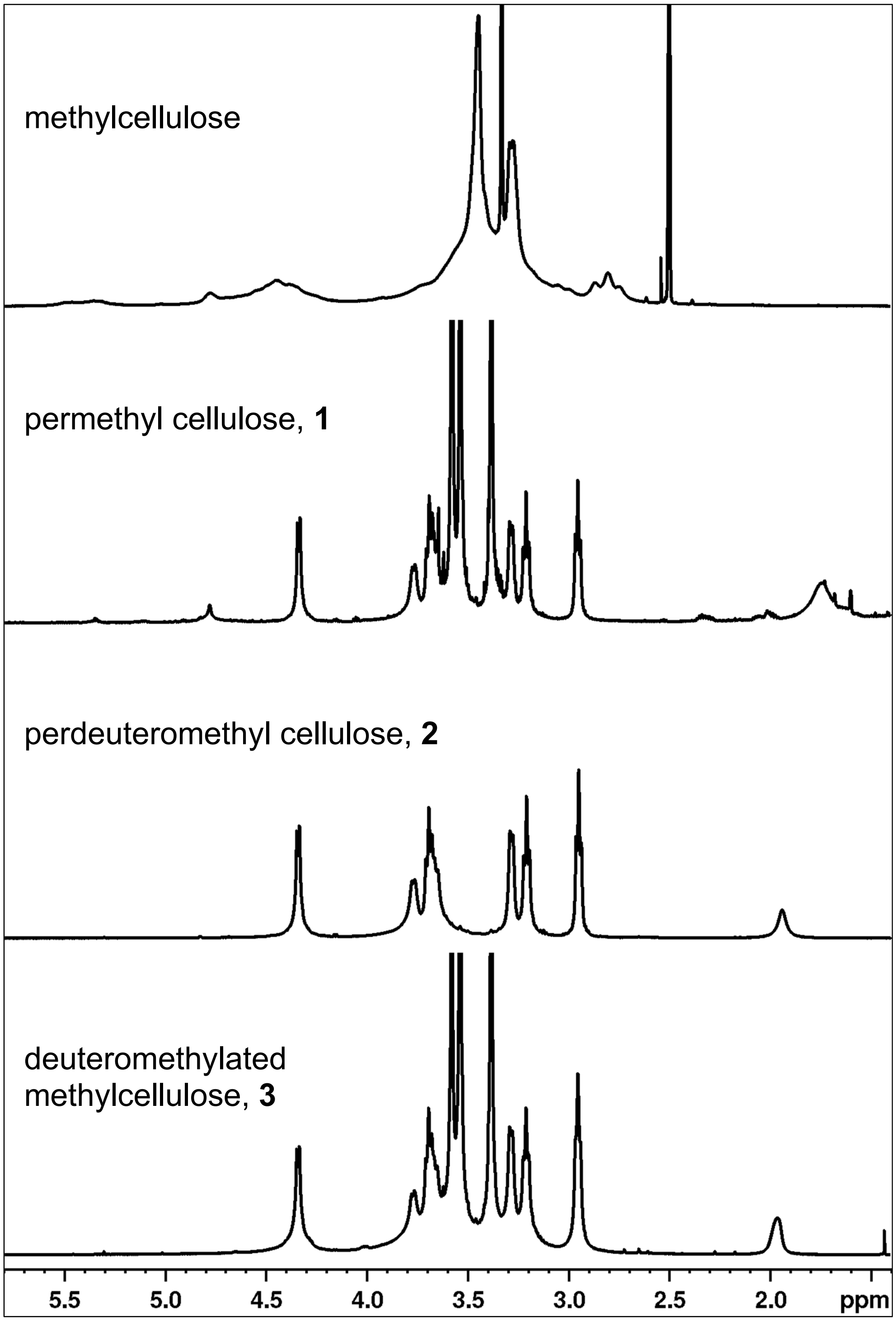

Fig. S2 ^{1}H-NMR spectra of **1**, **2**, **3** (600 MHz, $CDCl_3$), and MC for comparison (600 MHz, DMSO-d_6)

1.3. ESI-MS of 1 and 2

1.3.1. ESI-MS of 1

Nearly 3 mg of **1** was partially hydrolyzed by 1 M trifluoroacetic acid (TFA) in a 1 mL V vial for 30 min at 120 °C. Repeated co-evaporation with toluene was performed at 22 °C under a stream of nitrogen to remove the TFA and dry the sample. After dilution to 10^{-4} M in LC-MS grade MeOH and filtration through a syringe PTFE membrane filter 0.45 µm, the sample was analyzed by ESI-MS in positive ion mode.

Electrospray ionization ion-trap mass spectrometry (ESI-IT-MS) was performed with an HCT Ultra ETDII (Bruker Daltonics, Bremen, Germany). The spectra were evaluated by Data Analysis 4.0 (Bruker Daltonics, Bremen, Germany). The sample was directly infused to the ESI source at a flow rate of 200 µL h−1. Nitrogen was used as dry gas (4 L/min, 300 °C) and as nebulizer gas (10 psi). Other instrumental parameters were as follows: capillary voltage -4500 V, endplate offset voltage -500 V, smart target 100,000, target mass 1000, positive ion mode. The spectrum is an average of 200 scans.

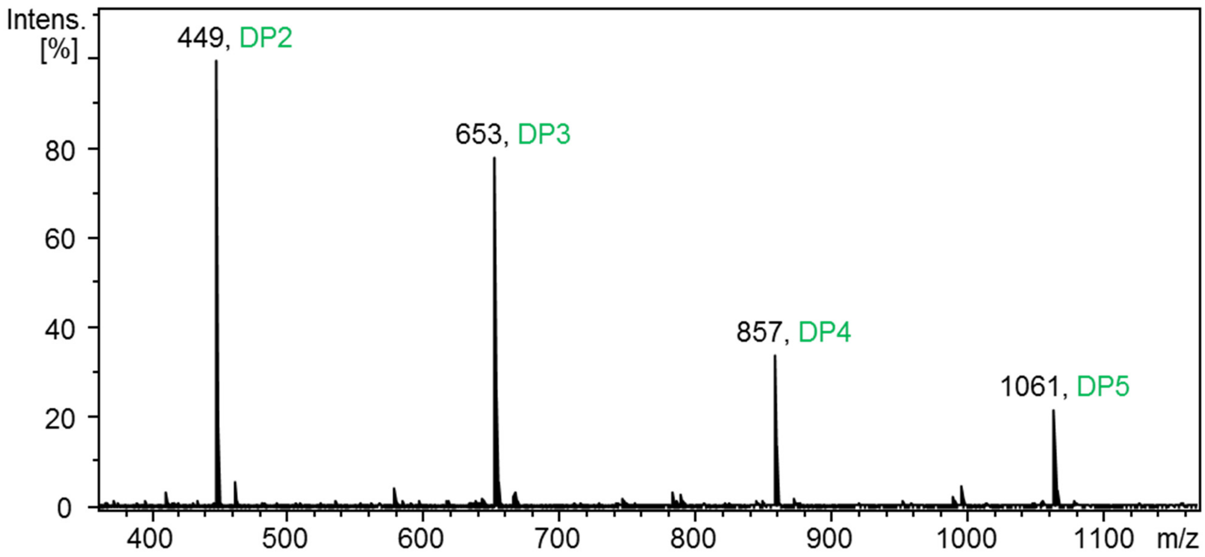

Fig. S3 DP2-5 cutout of the ESI-MS spectrum of partially hydrolyzed **1**; positive mode, $[M+Na]^+$ is detected

1.3.2. ESI-MS of 2

Nearly 3 mg of **2** was partially hydrolyzed as explained above for **1**. Thereafter, partially hydrolyzed products were labeled with *m*-aminobenzoic acid by reductive amination in MeOH as described in the experimental section of the manuscript. After dilution to 10^{-4} M in LC-MS grade MeOH and filtration through a syringe PTFE membrane filter 0.45 µm, the sample was analyzed by ESI-MS in negative ion mode.

ESI-IT-MS was performed by an HCT Ultra ETDII (Bruker Daltonics, Bremen, Germany). The spectra were evaluated by Data Analysis 4.0 (Bruker Daltonics, Bremen, Germany). The sample was directly infused to the ESI source at a flow rate of 200 µL h^{-1}. Nitrogen was used as dry gas (5 L/min, 300 °C) and as nebulizer gas (10 psi). Other instrumental parameters were as follows: capillary voltage 3500 V, endplate offset voltage -500 V, smart target 100,000, target mass 1000, negative ion mode. The spectrum is an average of 150 scans.

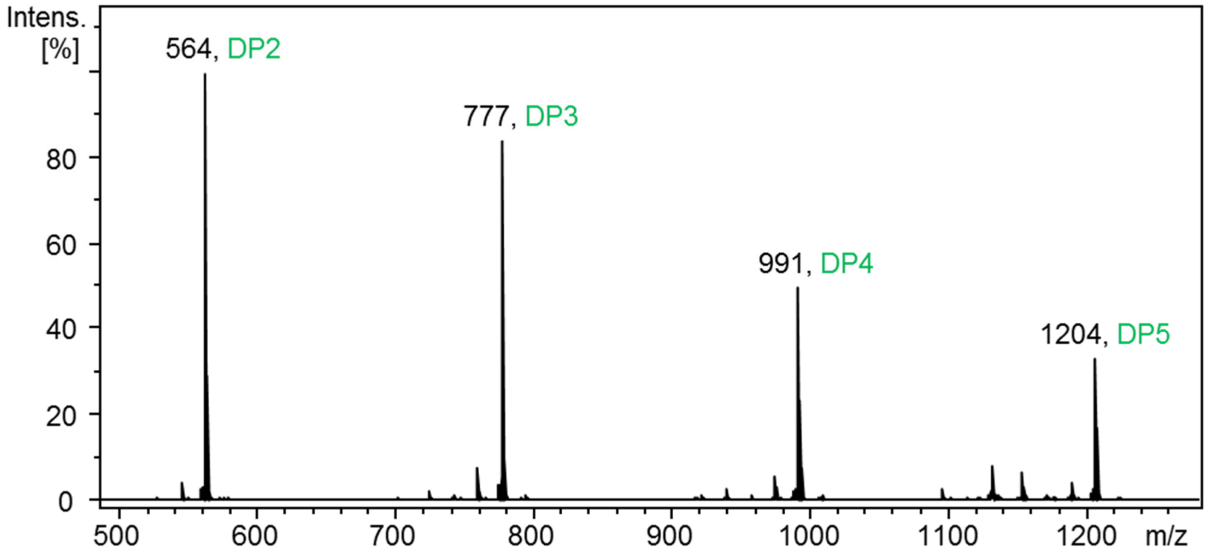

Fig. S4 DP2-5 cutout of the ESI-MS spectrum of partially hydrolyzed and *m*-ABA labeled **2**; negative mode, $[M-H]^-$ is detected

1.4. Oligomer analysis of 3 by LC-MS

Sample preparation and LC-MS analysis of **3** was performed as explained in the experimental section of the manuscript.

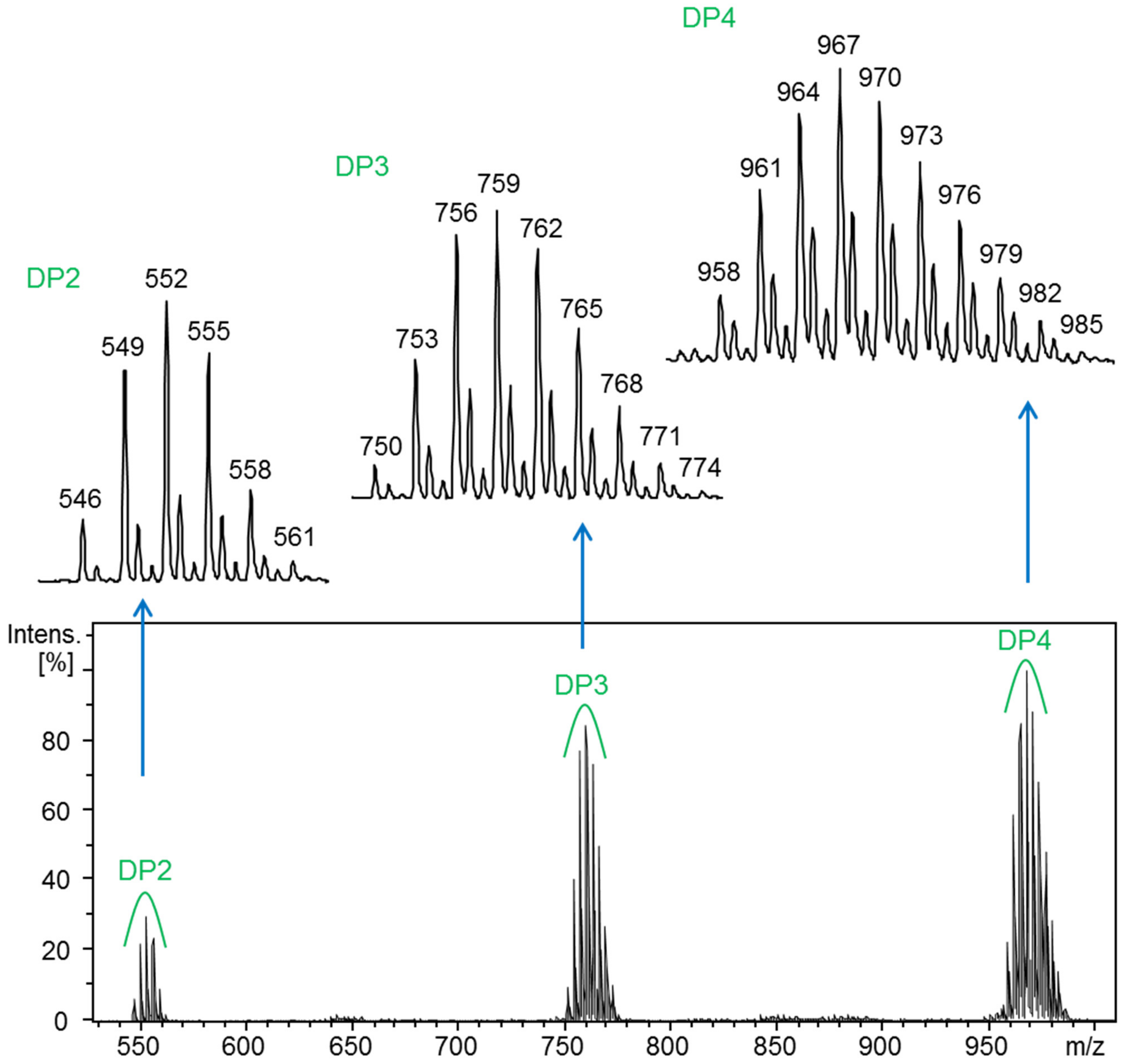

Fig. S5 DP2-4 cutout of the LC-MS spectrum of **3**

Evaluated methyl profiles of **3** (DP2-7) based on the LC-MS results are shown in Fig. S6.

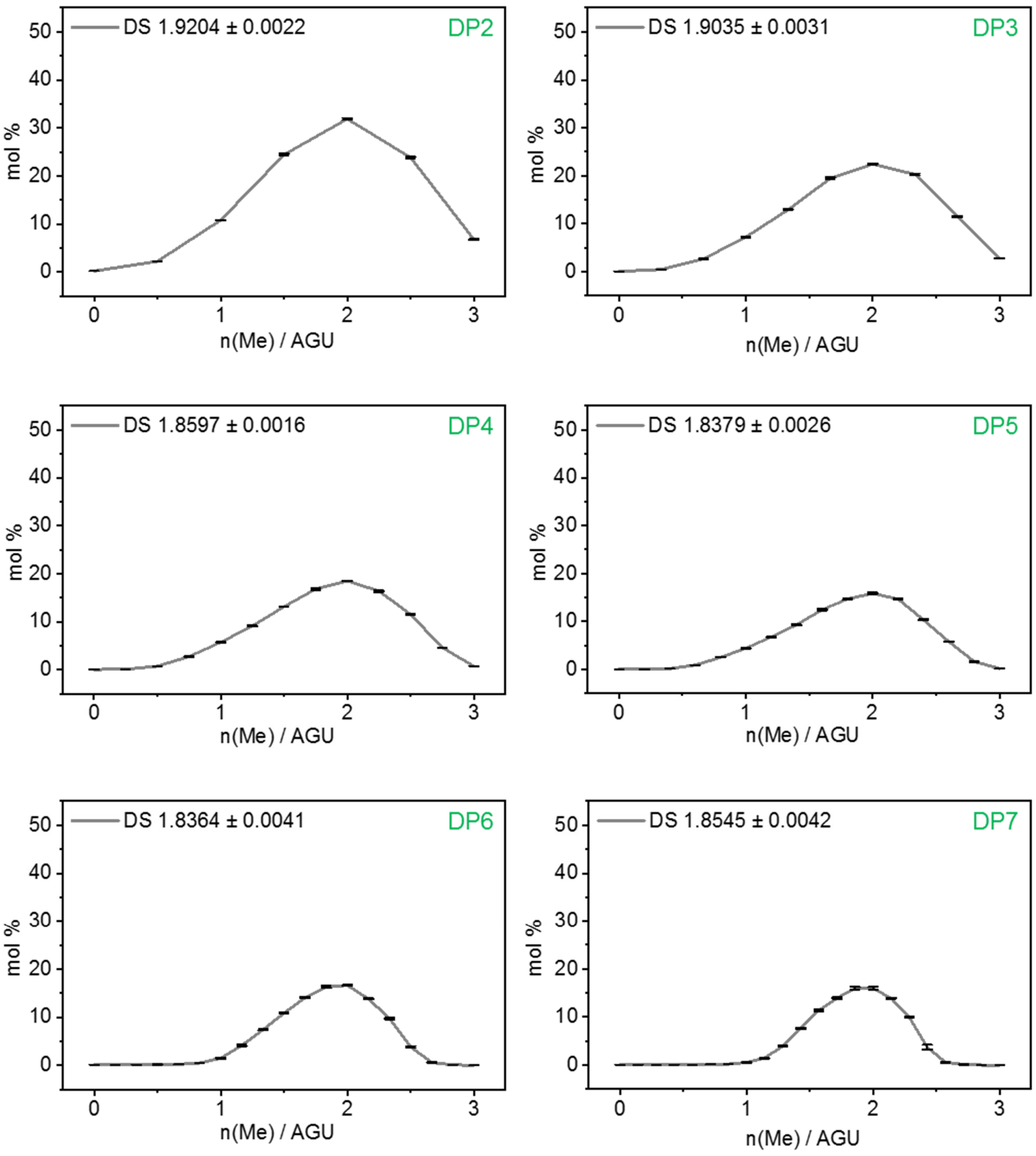

Fig. S6 Methyl profile and the DS values of **3** (DP2-7) evaluated from the LC-MS results. Values are the averages of three times measurement of three independent samples (total of 9 measurements)

1.5. Monomer analysis of MC by GC-FID chromatography

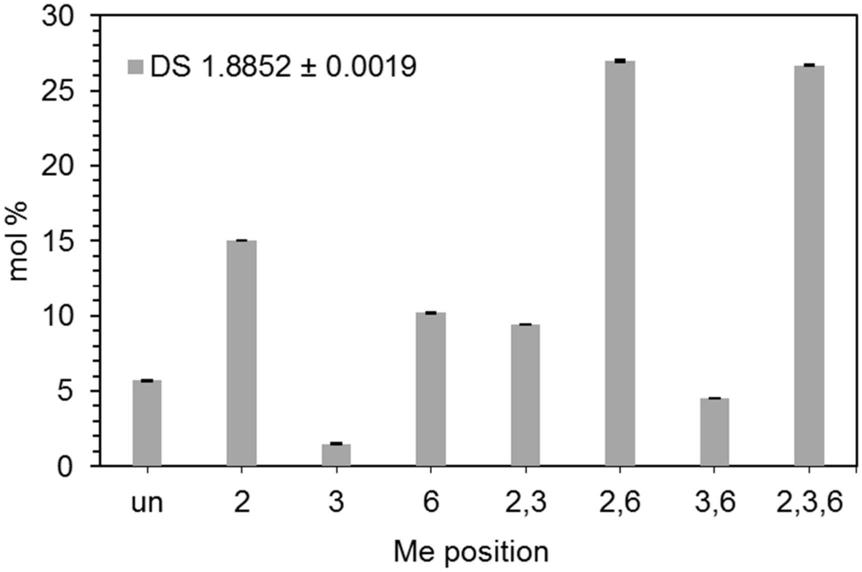

Fig. S7 Evaluated monomer analysis of MC after total hydrolysis, alditol acetate formation, and analysis by GC-FID chromatogram. Values are the averages of three measurements of five independent samples. Experimental details are explained in the experimental section of the manuscript

2. ATR-FTIR and ^{1}H-NMR of transglycosylation-a products

2.1. ATR-FTIR of transglycosylation-a

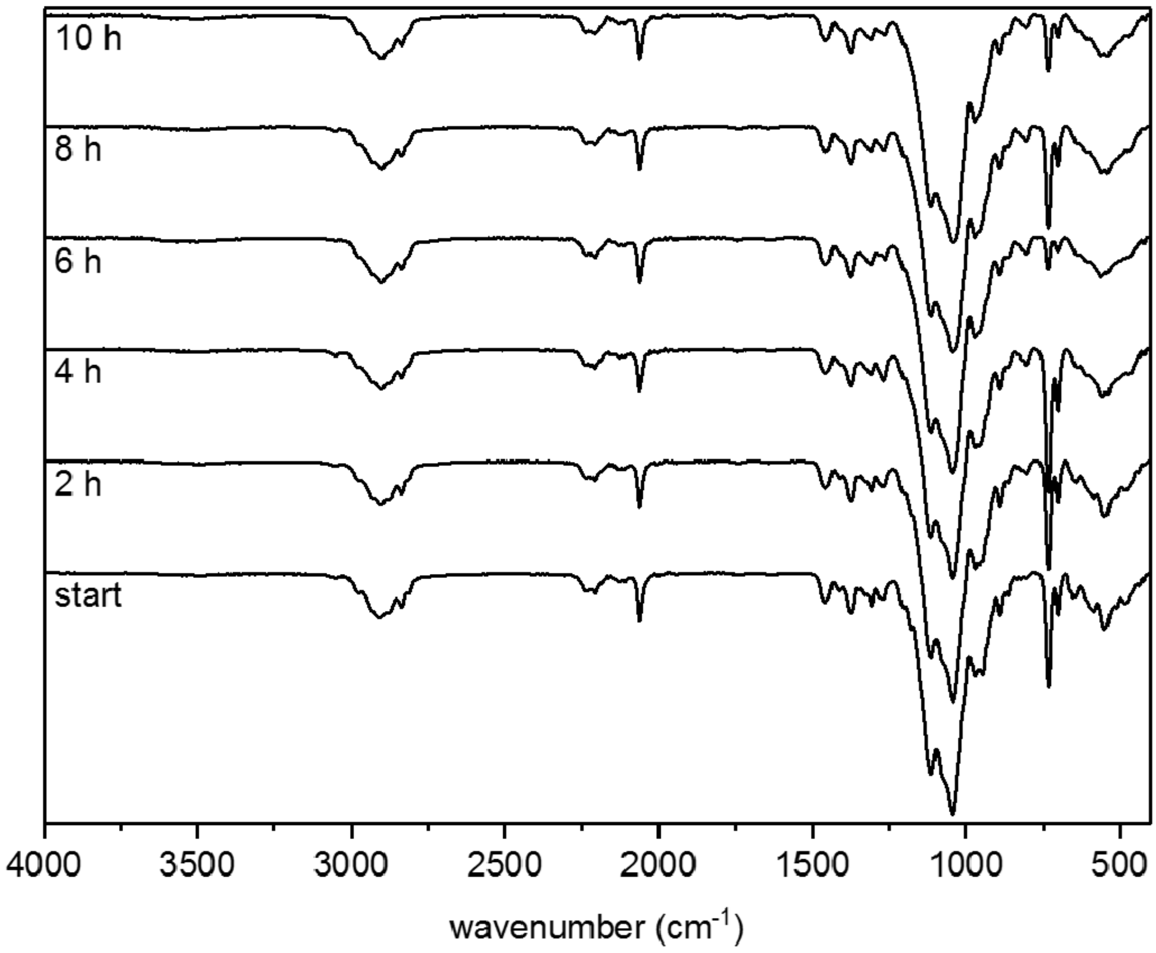

Fig. S8 ATR-FTIR spectra of transglycosylation-a products

2.2. ^{1}H-NMR of transglycosylation-a

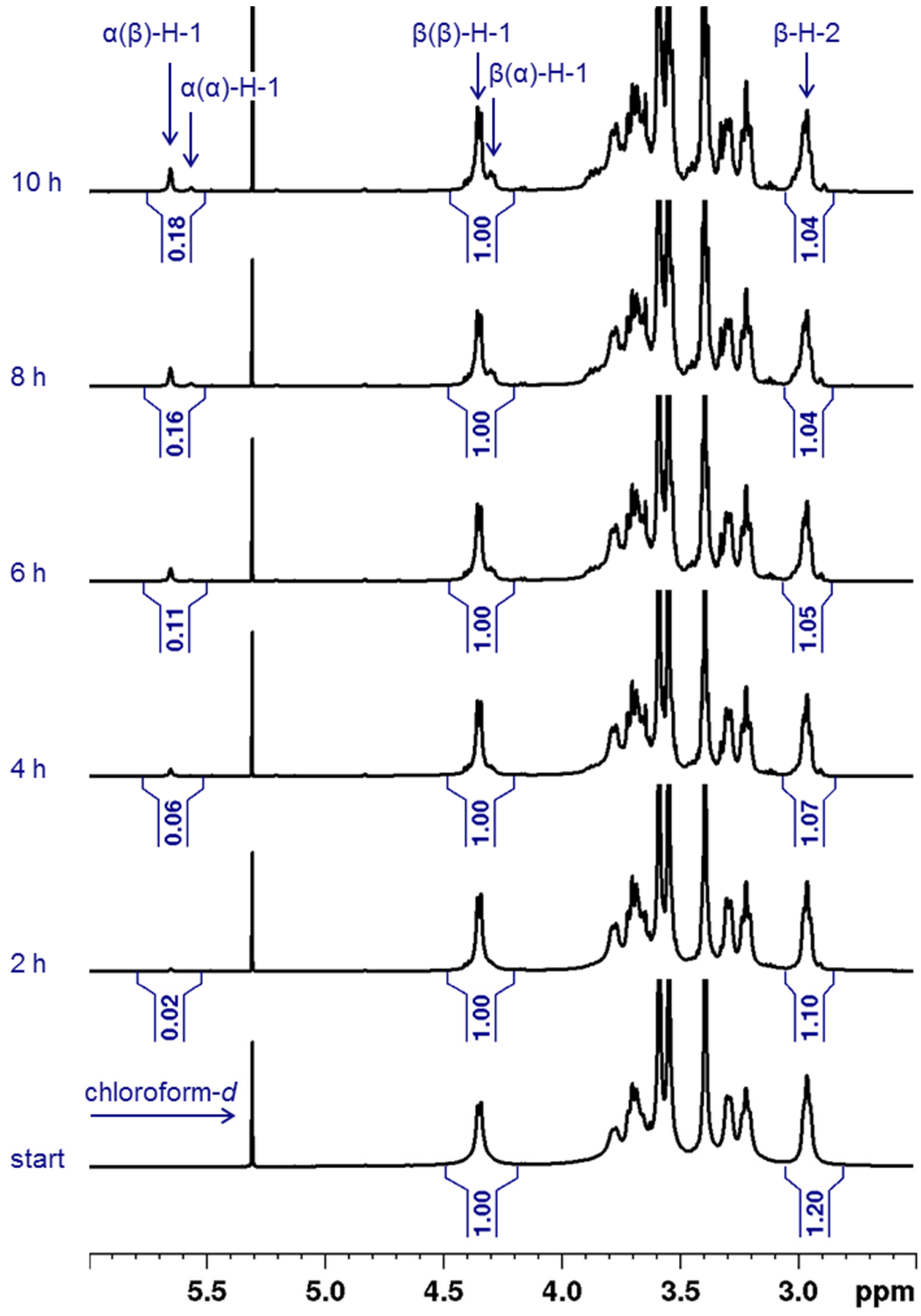

Fig. S9 ^{1}H-NMR spectra of transglycosylation-a products (600 MHz, $CDCl_3$)

3. The influence of the scan speed on ESI-MS analysis of transglycosylation-a products

In order to investigate whether the scan speed plays a role in mass spectrometry analysis and subsequent average block length (BL) evaluations, each transglycosylation-a product was analyzed 6 times by ESI-MS; 3 times by the standard-enhanced mode (8100 *m/z* /s) and 3 times by ultra-scan mode (26000 *m/z* /s). Other instrumental parameters were the same and as explained below. BL of the products were evaluated based on the results at DP2 and DP3 level (Fig. S10).

The sample was directly infused to the ESI source at a flow rate of 200 µL h^{-1}. Nitrogen was used as dry gas (5 L/min, 300 °C) and as nebulizer gas (10 psi). Other instrumental parameters were as follows; negative ion mode, capillary voltage 3500 V, endplate offset voltage -500 V, smart target 100,000, target mass 1000, scan range *m/z* 500-1300, number of scans 200.

The following equations were used for calculation of the average block length (BL) based on DP2 and DP3 results [1].

$$\mathrm{BL_{DP2}} = \frac{\mathrm{Int.}\,(m/z\,546) + \mathrm{Int.}\,(m/z\,564)}{\mathrm{Int.}\,(m/z\,555)} + 1$$

$$\mathrm{BL_{DP3}} = \frac{[\mathrm{Int.}\,(m/z\,750) + \mathrm{Int.}\,(m/z\,777)] + 0.5[\mathrm{Int.}\,(m/z\,759) + \mathrm{Int.}\,(m/z\,768)]}{0.5[\mathrm{Int.}\,(m/z\,759) + \mathrm{Int.}\,(m/z\,768)]} + 1$$

[1] Adden, R.; Bösch, A.; Mischnick, P. Novel Possibilities by Cationic Ring-Opening Polymerisation of Cyclodextrin Derivatives: Preparation of a Copolymer Bearing Block-Like Sequences of Tri-*O*-methylglucosyl Units. *Macromol. Chem. Phys.* **2004**, *205*, 2072–2079.

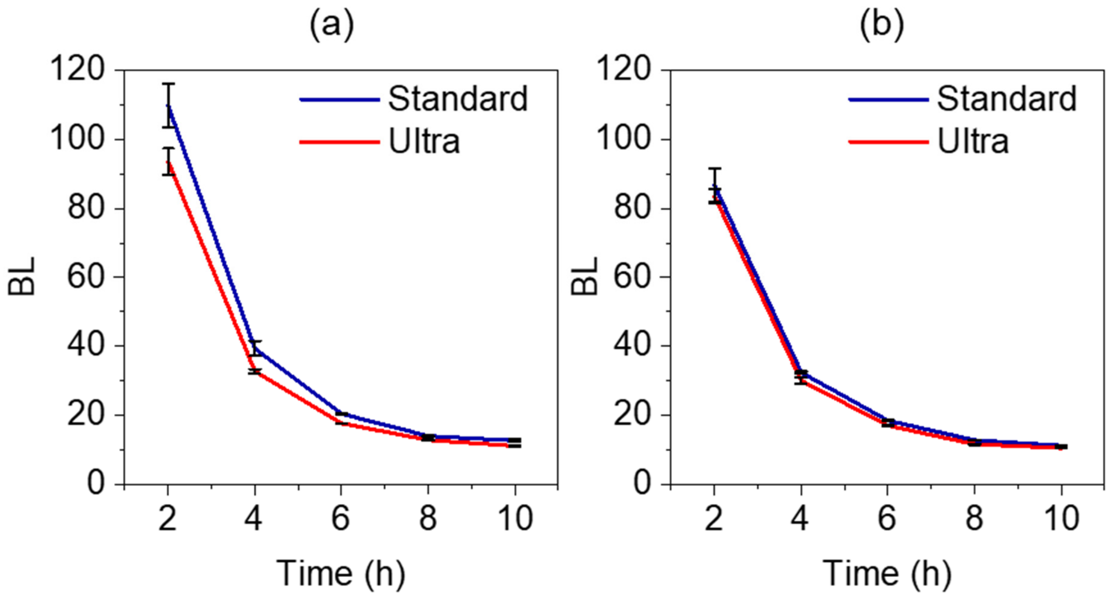

Fig. S10 Average block length (BL) of Trg-a products based on (a) DP2, and (b) DP3 results of ESI-MS at different scan modes. Standard refers to the standard-enhanced mode (8100 *m/z* /s) and ultra refers to the ultra-scan mode (26000 *m/z* /s). Values are the averages of three times measurement of each sample

Comparison of the data illustrated in Fig. S10 shows that:

- The difference between the evaluated average block lengths at different scan speeds is more pronounced in case of DP2 than DP3
- Measurement uncertainties are slightly better for measurements by ultra-scan mode

Further investigation of the influence of scan speed on quantitative ESI-MS- and LC-MS analysis of glucan derivatives is in progress in our lab.

4. ESI-MS^n of transglycosylation-a at the reaction time of 10 h

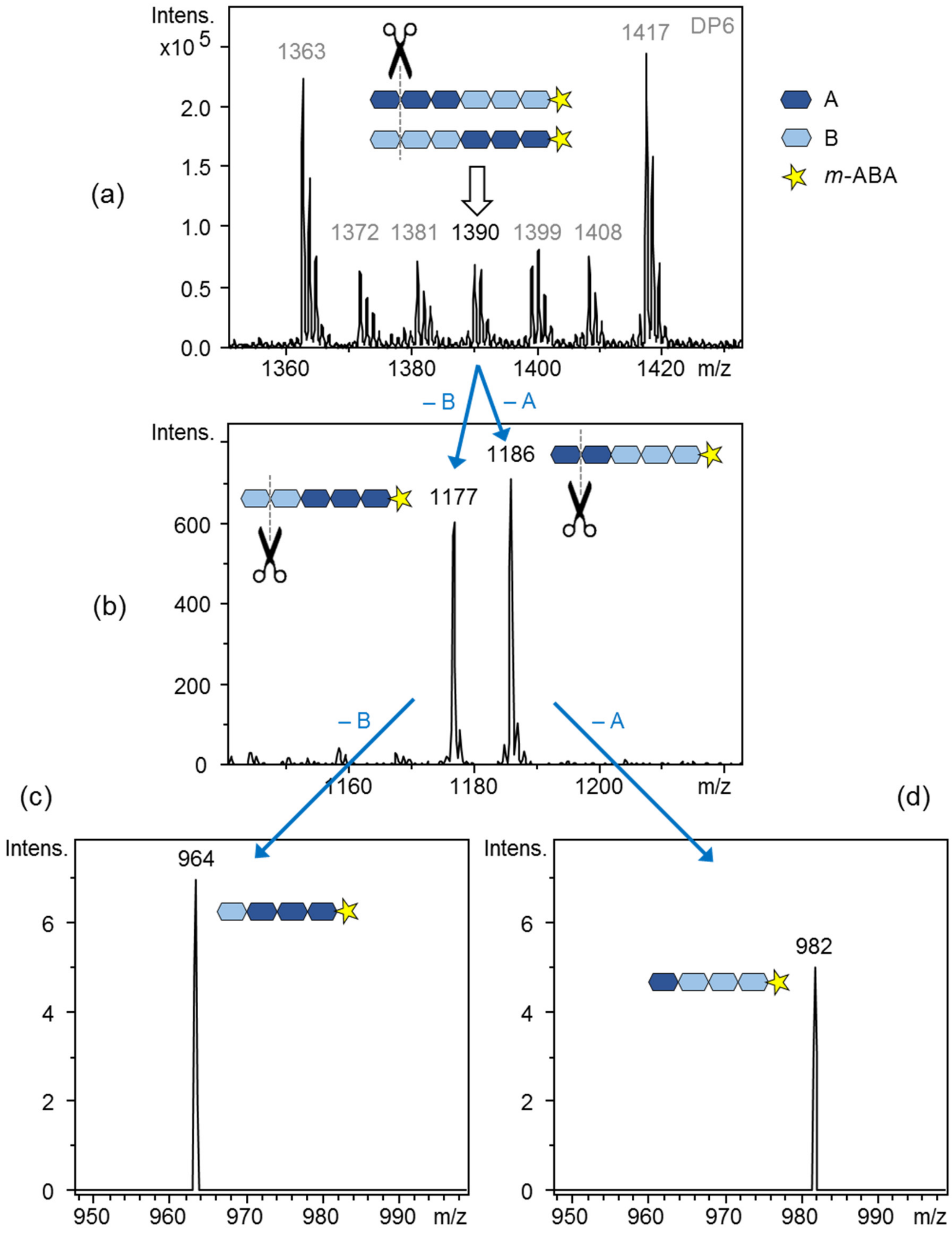

Fig. S11 DP6 cutout of ESI-MS spectra of transglycosylation-a after 10 h (a), as well as the CID-MS^2 (b), and CID-MS^3 (c, d) of the indicated peaks

5. Me-profiles of transglycosylation-b products

5.1. Me-profile of the transglycosylation-b product after 10 h

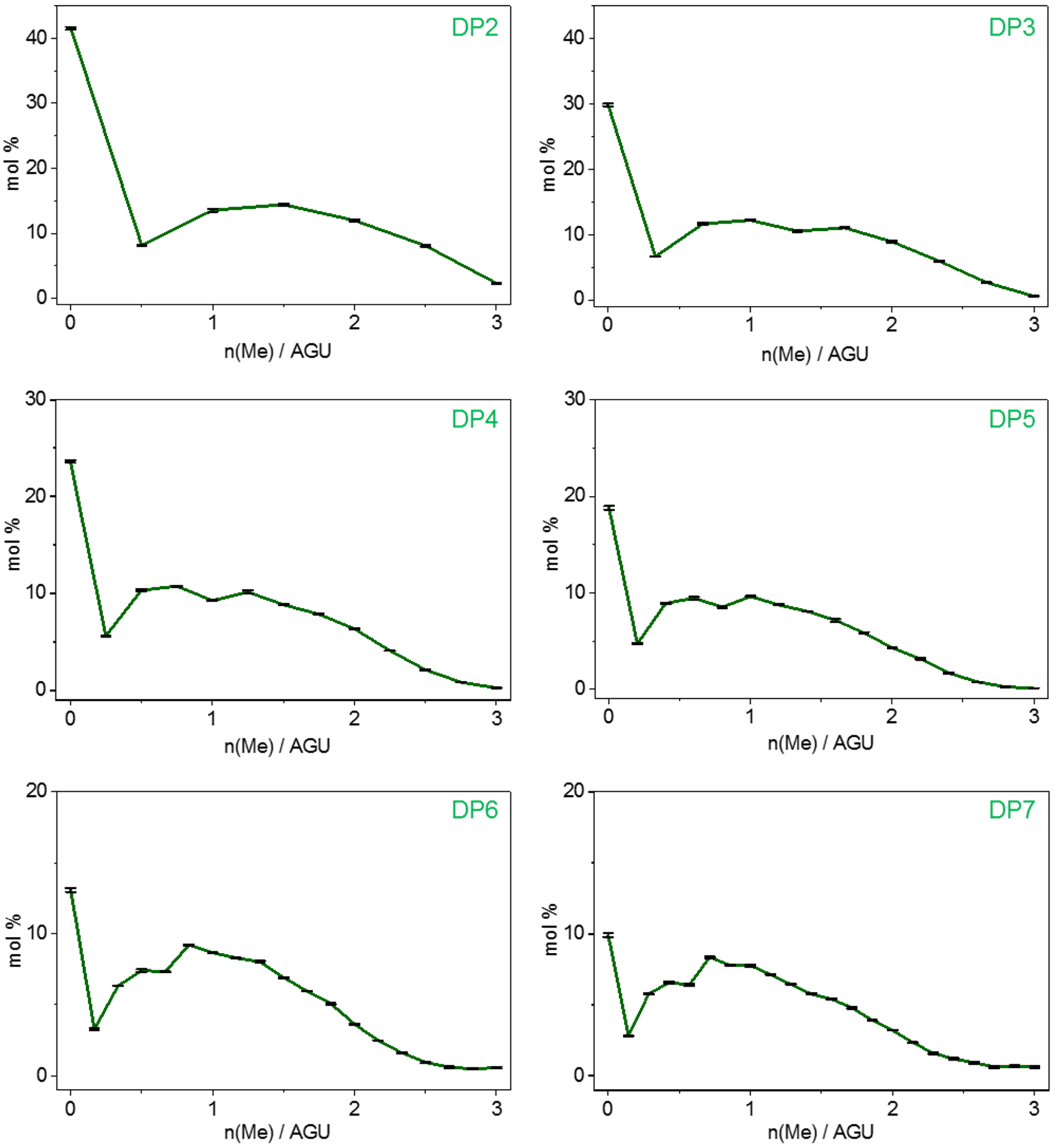

Fig. S12 Evaluated Me-profiles of Trg-b products after 10 h (DP2-7). Different scales of y-axes are used for a better demonstration of the polymodality of Me-profiles. Values are the averages of three times measurement of each sample

5.2. Subtraction of *m/z* ±17 components from the Me-profile

In order to eliminate the contribution of under-deuteromethylated components of the products from the Me-profile, LC-MS results were re-evaluated as follow:

1. Mass spectra were re-produced by the integration of the total ion current chromatogram in a way that the under/over-deuteromethylated domains of each DP were excluded as much as possible. However, due to the complexity of the LC-MS data, as explained in the manuscript, it was not possible to completely exclude them. Therefore,
2. The contribution of *m/z* ±17 peaks of the remaining under/over-deuteromethylated components and their isotopic peaks were subtracted

The results of this modification are presented in Fig. S13b and compared to the Me-profiles prior to this modification (Fig. S13a).

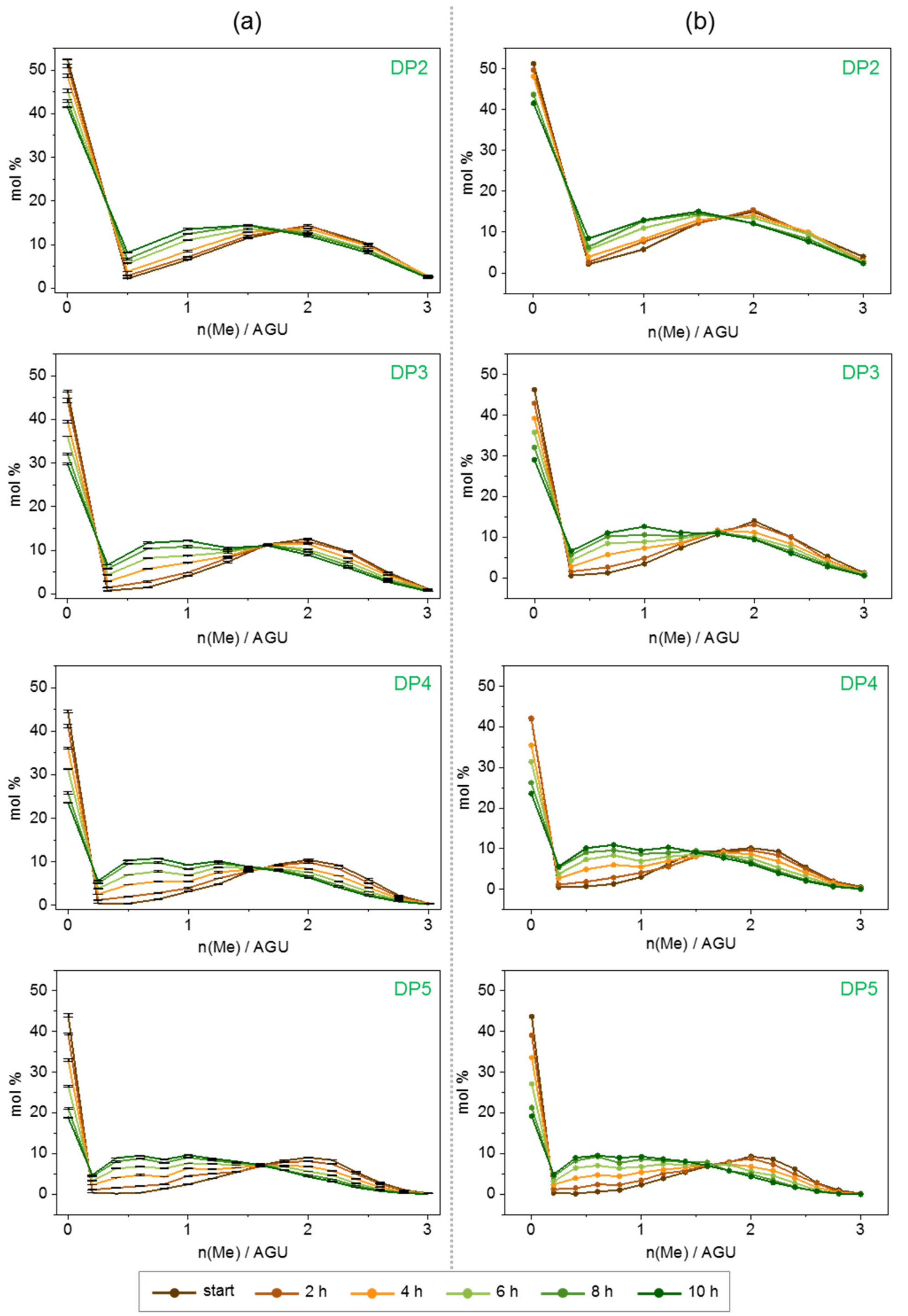

Fig. S13 Comparison of the Me-profiles of Trg-b products at DP2-5 level before (a), and after subtraction of the contribution of m/z ±17 components from the Me-profiles (b). Values demonstrated in Fig. S13a are the averages of 3 times measurement of each sample whereas those in Fig. S13b are based on one-time measurement of the samples

5.3. A model for Me-profile of transglycosylation products over time

To obtain a model for the Me-profile of transglycosylation-b products over the reaction time based on DP5 results shown in Fig. 9 of the manuscript, the relative amounts of n(Me)/AGU of DP5 were separately plotted against the reaction time (Fig. S14).

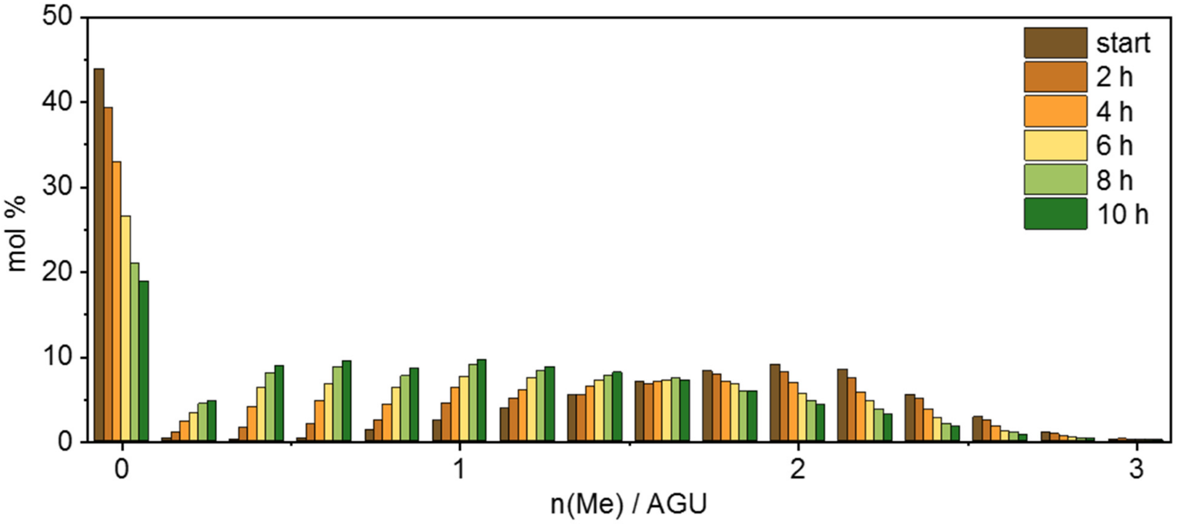

Fig. S14 The changes of each n(Me)/AGU of DP5 over the reaction time

Thereafter, the changes of each n(Me)/AGU were separately fitted by the non-linear Boltzmann sigmoidal fitting feature of Origin-Pro 2018 (Northampton, Massachusetts, USA). Boltzmann sigmoidal formula and the corresponding fitting parameters for each n(Me)/AGU are presented in the following equation and Table S1, respectively (y is the mole percent of each n(Me)/AGU, and x is the reaction time).

$$y = \frac{A_1 - A_2}{1 + e^{(x-x_0)/dx}} + A_2$$

By plotting the obtained fitting curve of each n(Me)/AGU against the reaction time, the model shown in Fig. 10 of the manuscript is obtained.

Table S1 Parameters of Boltzmann sigmoidal fitting for each n(Me)/AGU of DP5

		Boltzmann fitting parameters			
		A_1	A_2	x_0	dx
n(Me)/AGU	0.00	47.4318	16.6433	4.2492	2.1670
	0.20	-0.4236	5.1158	4.0863	2.2808
	0.40	-0.9539	9.4163	4.1974	1.9838
	0.60	-1.4241	10.2320	3.9317	2.2843
	0.80	0.5004	8.9896	4.4352	2.0187
	1.00	-7.1466	11.2074	-0.3988	4.3950
	1.20	1.6508	9.4313	2.8125	3.0494
	1.40	5.0441	8.2837	4.5761	2.0621
	1.60	6.8145	7.2064	4.1006	0.8114
	1.80	8.4970	5.6231	4.5197	2.0578
	2.00	9.9002	3.9360	3.8851	2.2051
	2.20	9.9103	2.5624	3.5643	2.6155
	2.40	5.9927	1.5042	4.0769	1.9182
	2.60	3.1922	0.7364	3.3628	1.7474
	2.80	1.4563	0.1887	2.2286	2.5293
	3.00	0.1780	0.1010	4.0000	0.0004M
